# Immunotherapy in Asia: current insights and prospects in Southeast Asia and Malaysia

**DOI:** 10.3389/fonc.2026.1847284

**Published:** 2026-08-13

**Authors:** Ros Suzanna Ahmad Bustamam, Yee Peng Phoon

**Affiliations:** 1 Department of Radiotherapy and Oncology, Hospital Kuala Lumpur, Kuala Lumpur, Malaysia; 2 Center for Immunotherapy & Precision Immuno-Oncology, Department of Cancer Sciences, Cleveland Clinic, Cleveland, OH, United States; 3 School of Medicine, Case Western Reserve University, Cleveland, OH, United States

**Keywords:** antibody, biomarker, cancer immunotherapy, car-t, cell therapy, Malaysia, regulation, Southeast Asia

## Abstract

Cancer immunotherapy has transformed the global oncology landscape, offering durable responses across malignancies once considered untreatable. In Asia and Southeast Asia, regions facing a rising cancer burden, countries such as Malaysia are progressively integrating immunotherapeutic approaches, including immune checkpoint inhibitors and CAR-T cell therapies. Regional progress is supported by investments in research infrastructure, expanding clinical trial participation, and cross-border regulatory collaboration. However, significant challenges persist. Diverse genetic and epidemiological profiles complicate treatment generalizability, while disparities in healthcare access, limited biomarker validation, and high costs restrict widespread implementation, particularly in low- and middle-income settings. In Southeast Asia, including Malaysia, these challenges are intensified by urban-rural disparities in healthcare access and financial limitations. However, emerging opportunities such as biomarker-informed precision immunotherapy, artificial intelligence-enabled treatment personalization, expanded telemedicine services, and enhanced access frameworks offer promising pathways forward. This review highlights the current landscape, ongoing challenges, and future prospects of cancer immunotherapy in Asia and Southeast Asia, with a particular focus on Malaysia’s growing role in this evolving field. Cross-regional cooperation, regulatory harmonization, and personalized treatment strategies are crucial for equitable access to cancer therapies across this region. By synthesizing fragmented and underrepresented evidence from Southeast Asia within a broader Asia-focused immunotherapy framework, this review aims to provide a consolidated reference that can guide future research prioritization, strengthen regional policy development, and support the design of more inclusive and scalable immunotherapy implementation strategies.

## Graphical Abstract

**Figure:**
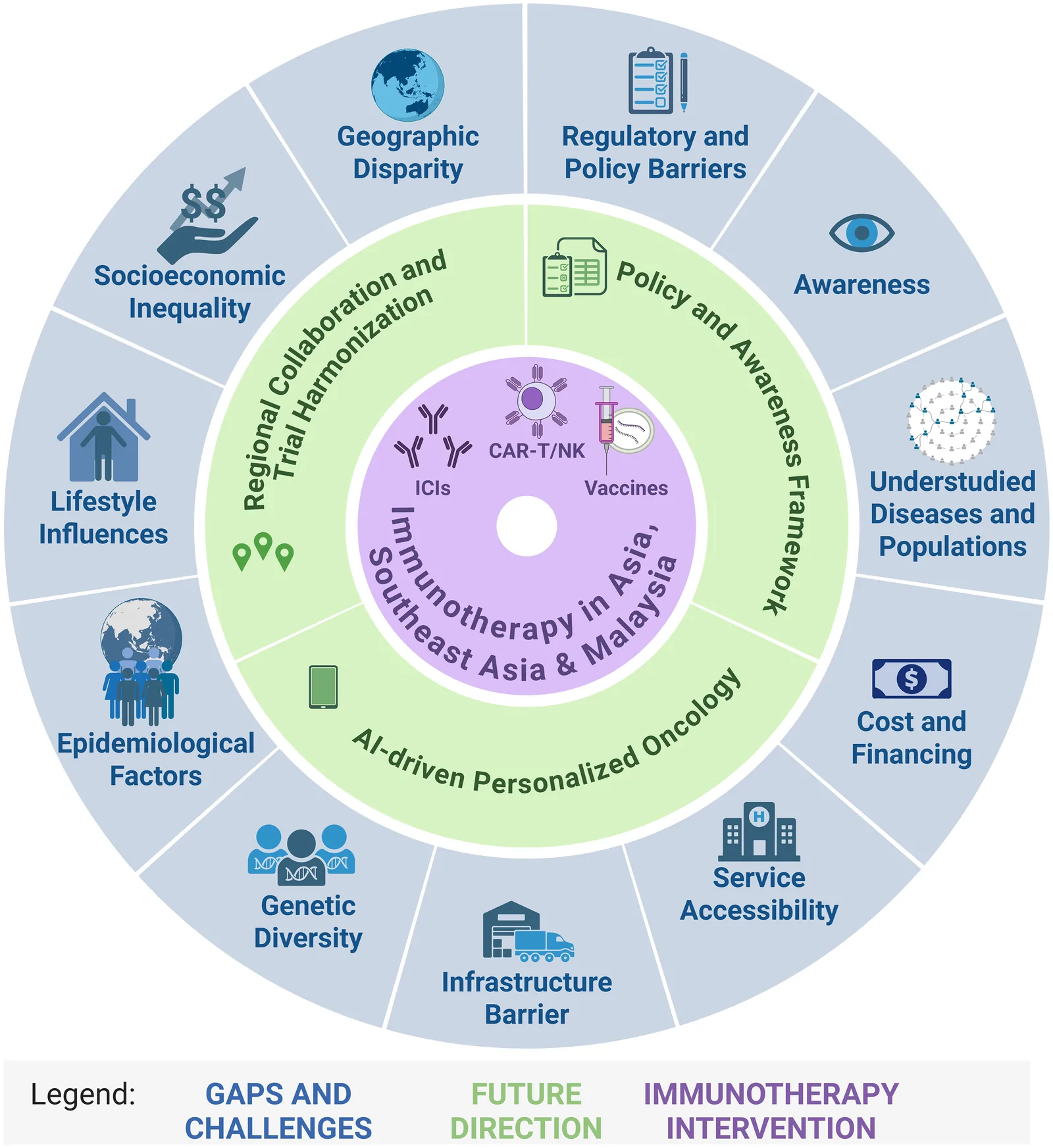
Created in BioRender. Phoon, Y. (2026) https://BioRender.com/zuvd35l

## 1 Introduction

**Figure 1:**
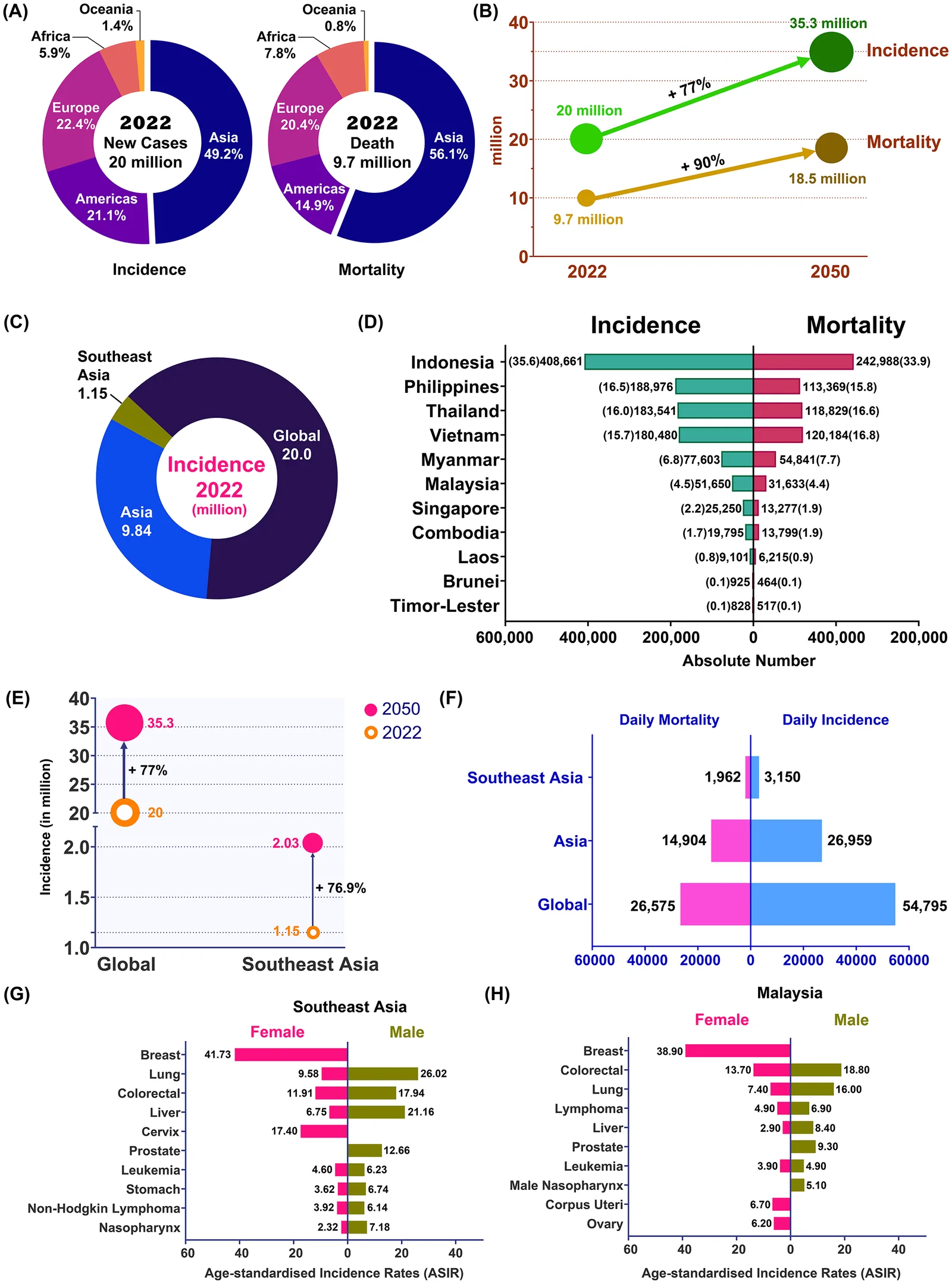
Cancer Statistic. **(A)** Newly diagnosed cancer cases and related mortality worldwide in 2022. **(B)** Projected global cancer incidence and mortality estimates for 2050. **(C)** Cancer incidence in 2022, comparing global, Asia and Southeast Asia. **(D)** Country-specific cancer incidence and mortality in Southeast Asia (rates shown as percentages). **(E)** Estimated new cancer cases in Southeast Asia by 2050. **(F)** Daily cancer incidence and mortality. **(G)** Top ten cancers in Southeast Asia by gender. **(H)** Top ten most cancers in Malaysia by gender. Source: GLOBOCAN 2022, Dee et al. and MNCR 2017-2021.

Cancer remains a leading cause of morbidity and mortality worldwide, with 20 million newly diagnosed cases and 9.7 million deaths in 2022. Asia bears a disproportionate share, accounting for nearly 50% of global cases and 56% of deaths (1) (**Figure 1A**). Global incidence is projected to rise to 35 million cases by 2050 (1), a 77% increase from 2022, with deaths reaching 18.5 million, a 90% increase (**Figure 1B**). Southeast Asia contributed approximately 1.15 million new cases in 2022 (5.6% of the global burden) (2) (**Figures 1C, D**), expected to rise to 2.03 million by 2050 (1, 2), a 76.9% increase (**Figure 1E**). Daily, around 55, 000 new cancer cases occur worldwide, including 27, 000 in Asia and 3, 000 in Southeast Asia (**Figure 1F**), with nearly 50% resulting in death, translating to approximately 26, 575 global deaths daily, 15, 000 in Asia and 2, 000 in Southeast Asia (1, 2) (**Figure 1F**).

**Figure 2:**
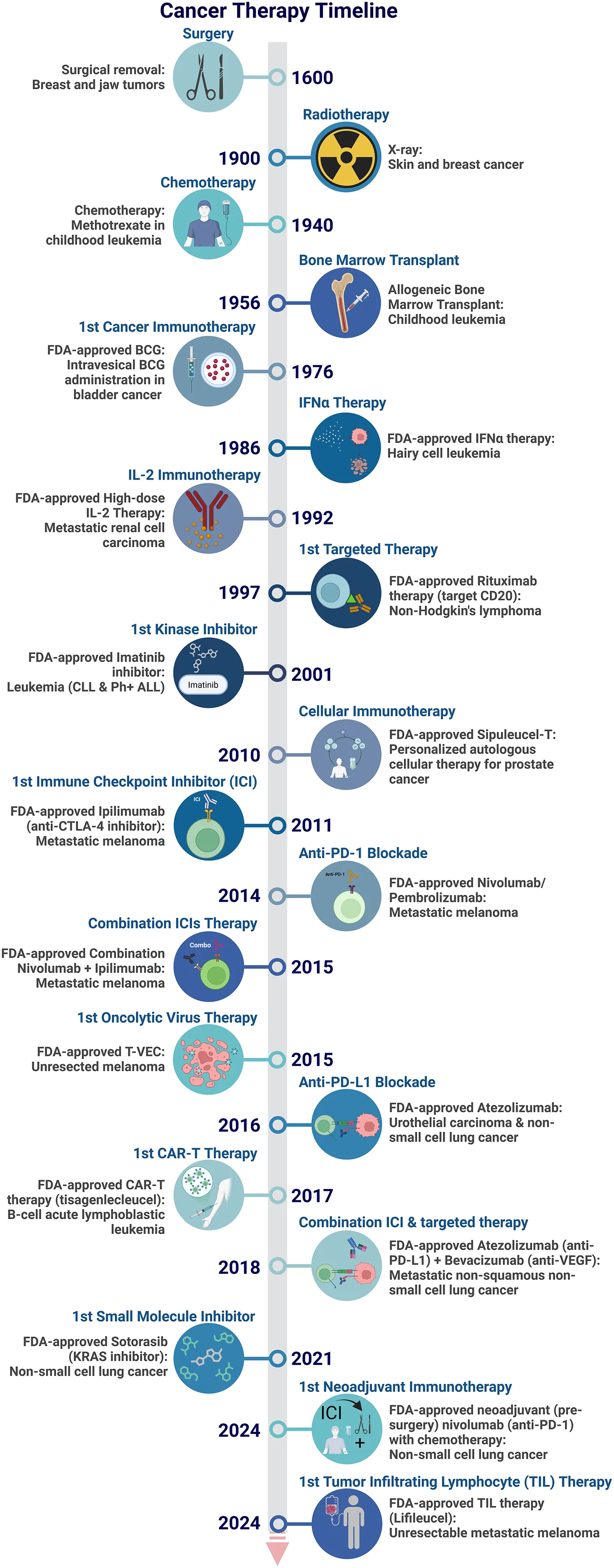
Timeline of cancer therapy development. timeline of selected milestones of cancer therapy development. Source: Zhang *et.al.*, Liu *et.al.*, Masucci *et.al.*, Nurieva *et.al.*, Wang et al. Created in BioRender. Phoon, Y. (2026) https://BioRender.com/sdknmkl.

The most common cancers in Asia include lung, liver, breast, colorectal, stomach, oral and prostate cancers (3). In Southeast Asia, breast cancer is the most prevalent, followed by lung, colorectal, liver and cervical cancers (2). In Malaysia, the Malaysia National Cancer Registry Report 2017-2021(MNCR) reports the most frequent diagnoses as breast, colorectal, lung, lymphoma, liver, prostate, leukemia, nasopharynx, corpus uteri and ovarian cancers (4) (**Figures 1G, H**). With the rising burden of cancer and persistently poor outcomes, treatments have evolved from surgery, radiotherapy, and chemotherapy to targeted and immune-based therapies, transforming patient care (**Figure 2**).

Cancer immunotherapy has revolutionized oncology by harnessing the immune system to recognize and eliminate malignant cells. Over the past two decades, multiple immunotherapeutic strategies, including immune checkpoint inhibitors (ICIs), chimeric antigen receptor T-cell (CAR-T) and CAR-natural killer (CAR-NK) therapies, cancer vaccines, and antibody-based therapies, have demonstrated significant clinical benefits across diverse malignancies (5–7). ICIs have notably improved outcomes in metastatic melanoma and non-small cell lung cancer (NSCLC) (8–12), while combination approaches with chemotherapy, targeted therapies, or radiotherapy have enhanced efficacy and overcoming resistance (13–19). Early disease applications, including neoadjuvant immunotherapy, show promise in improving surgical outcomes and reducing recurrence (20–27). These developments represent a paradigm shift in cancer treatment, expanding the role of immunotherapy across disease stages and tumor types.

Despite these advances, the impact of immunotherapy in Asia and Southeast Asia is shaped by unique challenges. Although cancer incidence is lower than in Western countries, Asia accounts for over 56% of global cancer deaths (see **Figure 1A**), reflecting higher case-fatality rate (1, 28). In Southeast Asia, age-standardized incidence rates (ASIRs) are below global averages, yet age-standardized mortality rates (ASMRs) for many cancers, including head and neck malignancies, exceed those in high-income regions (1, 2, 28). Contributing factors include late-stage diagnosis, limited access to screening and molecular diagnostics, and restricted availability of advanced therapies. High burdens of infection-associated cancers, such as hepatocellular carcinoma (HCC) and nasopharyngeal carcinoma (NPC) (1, 3, 28–31), further exacerbate the regional challenge. Additional barriers include high treatment costs, limited infrastructure, regulatory hurdles, delayed screening, and underrepresentation in clinical trials (3, 4, 32–34). Genetic and ethnic diversity adds complexity, influencing treatment response and biomarker applicability (3, 35). Population-specific genetic variation can affect biomarker performance, highlighting the need for diverse biomarker discovery efforts to advance precision medicine and equitable care. Addressing these gaps is essential for region-specific immuno-oncology strategies.

This review examines the current landscape of cancer immunotherapy in Asia and Southeast Asia, with a focus on Malaysia, identifies barriers to broader adoption, and outlines future directions to improve access and reduce the regional cancer burden.

## 2 Literature search strategy and methodology

This narrative review provides an updated overview of the cancer immunotherapy landscape in Asia, with a particular focus on Southeast Asia and Malaysia, highlighting recent advances, implementation challenges, and future opportunities to improve access and reduce cancer burden. A literature search was conducted in PubMed using the keywords such as cancer immunotherapy, biomarker, clinical trial, CAR-T, tumor-infiltrating lymphocyte, antibody, artificial intelligence, cancer, oncology, Malaysia, Asia, Southeast Asia, disparity, heterogeneity, barriers, challenges, infrastructure, regulation, policy, and cancer statistics. Ongoing and completed clinical trials were identified through ClinicalTrials.gov, and additional relevant publications, guidelines, policy documents, and official reports were obtained through manual review of reference lists and authoritative sources. Studies and therapeutic agents were selected based on their relevance to the scope and objectives of this review. Withdrawn agents, terminated or withdrawn clinical trials, studies not yet recruiting, and retracted publications were excluded. All literature searches, clinical trial searches, and document retrieval were performed up to Jun 2026.

## 3 Advances in immune checkpoint inhibitors and antibody-based therapies

Over the past decade, ICIs and antibody-based therapies have become central to cancer treatment, offering potent and durable anti-tumor activity. These agents act through distinct yet often complementary mechanisms, from direct tumor cell targeting to modulation of the tumor immune microenvironment (TME). Their clinical application is increasingly guided by biomarker-stratified strategies, optimizing patient selection and therapeutic outcomes. This section highlights recent advances in ICIs and antibody-based therapies, with emphasis on agents in development or under evaluation in clinical trials across Asia and Southeast Asia.

### 3.1 Immune checkpoint inhibitors

**Figure 3:**
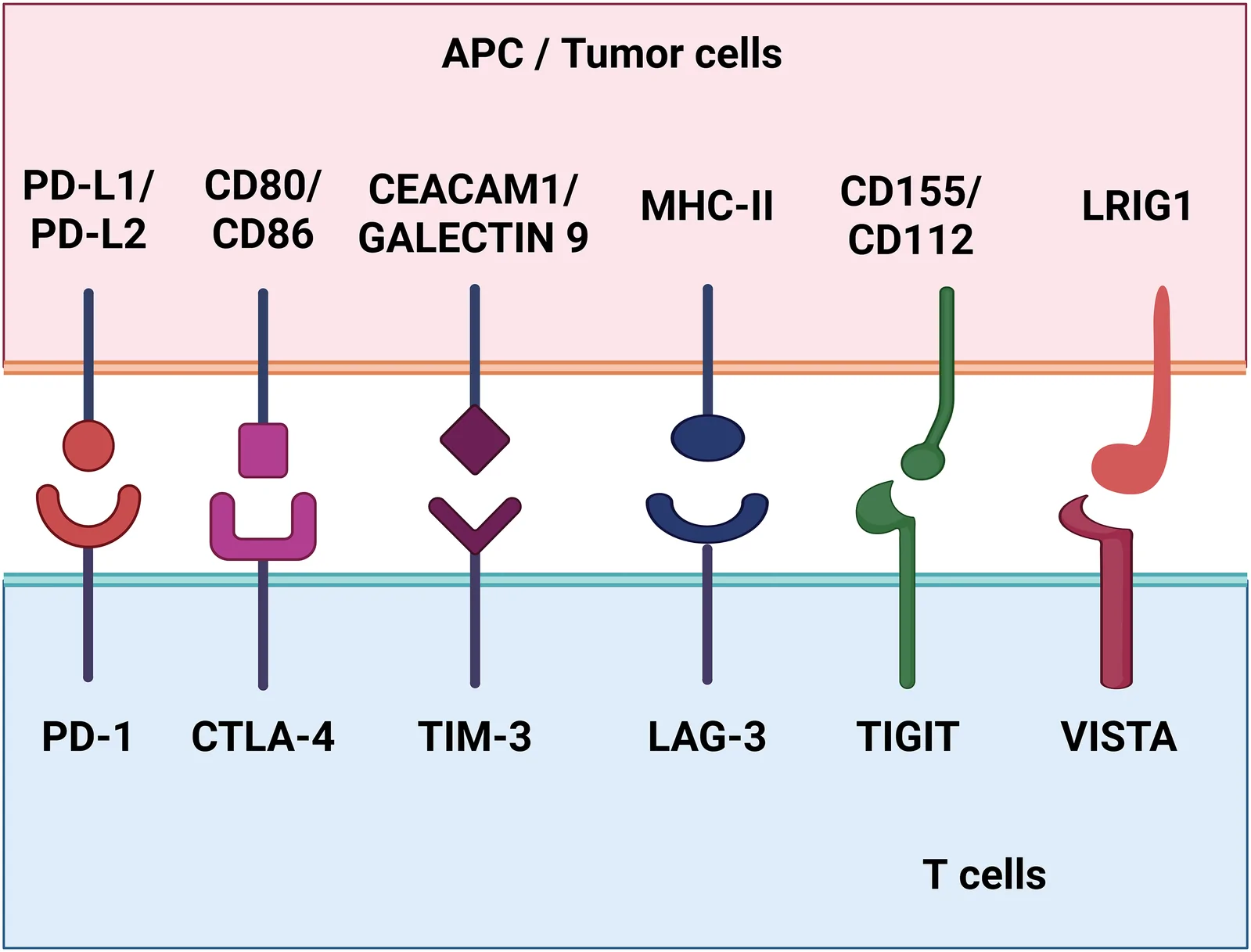
Inhibitory immune checkpoints. Inhibitory immune checkpoints and their corresponding ligands are depicted. Created in BioRender. Phoon, Y. (2026) https://BioRender.com/fvhlbzk.

Since the first U.S. Food and Drug Administration (FDA)-approved ICI, a variety of ICIs have been developed and continue to be evaluated to improve patient outcomes. These agents target regulatory pathways such as PD-1/PD-L1 and CTLA-4, which tumors exploit to evade immune detection. By blocking these inhibitory signals, ICIs restore T-cell activity and enhance anti-tumor immunity (36). Clinically, ICIs have demonstrated durable responses and improved survival across multiple cancers, including melanoma, lung, and renal cancers (37). Challenges remain, however, including variable response rates, identification of predictive biomarkers, and management of immune-related adverse events (AEs). Despite these hurdles, ICIs have become a cornerstone of modern oncology, driving advances in personalized cancer therapy. This section highlights ICIs that are currently approved, as well as novel agents under investigation or in development (**Figure 3**). The clinical trials summarized in **Supplementary Table 1** are organized by therapeutic agent class and country/region of conduct, with some NCT identifiers appearing across multiple entries due to evaluation of the same agents across different populations and study settings; the table is not exhaustive of all clinical trials conducted across the region.

#### 3.1.1 PD-1

PD-1 (Programmed Cell Death Protein 1) is an inhibitory receptor on activated immune cells that binds PD-L1/PD-L2 on tumors and antigen-presenting cells, suppressing T-cell activation. PD-1 functions mainly in peripheral tissues to maintain immune tolerance during the effector phase. Chronic PD-1 signaling in cancer drives T-cell exhaustion and immune evasion, while blockade of the PD-1/PD-L1 pathway restores effector function and synergizes with CTLA-4 inhibitors or chemotherapy. PD-1 stability is regulated by glycosylation and degradation, and alterations in the TME sustain its inhibitory signaling (38).

Anti-PD-1 antibodies, such as pembrolizumab and nivolumab, reinvigorate T-cell activity and have FDA approval for multiple malignancies, including NSCLC, melanoma, and urothelial carcinoma, with objective response rates (ORRs) of 20–40% (8, 9, 12, 22, 39–41). Combination checkpoint blockade enhances anti-tumor immunity and survival; in advanced melanoma, nivolumab plus ipilimumab achieved a 10-year median overall survival of 71.9 months and 96% melanoma-specific survival among patients progression-free at 3 year (12). Neoadjuvant PD-1 blockade, alone or combined with chemotherapy or other immunotherapies, shows promise in resectable NSCLC and melanoma, improving event-free survival (EFS) and major pathological response rates (e.g., KEYNOTE-671: 24-month EFS 62.4% vs 40.6% with chemotherapy alone) (42, 43).

In Asia, PD-1 inhibitors have shown promising efficacy in regionally prevalent cancers, including gastric, esophageal, nasopharyngeal, and lung cancers (44–48). A meta-analysis of phase III trials evaluating PD-1/PD-L1 inhibitors across multiple solid tumors, including non-small cell lung cancer (NSCLC), melanoma, renal cell carcinoma (RCC), head and neck squamous cell carcinoma (HNSCC), urothelial carcinoma, and other advanced cancers, suggested improved outcomes among Asian compared with non-Asian patients, with superior overall survival (OS) (hazard ratio [HR], 0.84; 95% confidence interval [CI], 0.75–0.94) and progression-free survival (PFS) (HR, 0.78; 95% CI, 0.59–0.97). However, these findings are mainly derived from *post hoc* subgroup analyses with heterogeneous trial designs and limited Asian representation. Potential explanations include differences in tumor biology, such as lower STK11 mutation rates in Asian versus non-Asian patients (1.6% vs 12.3% in OAK/POPLAR analyses), while pharmacokinetic differences remain uncertain (49).

In NSCLC, the phase III CheckMate 078 trial provided important evidence supporting PD-1 blockade in Asian populations. In a predominantly Chinese cohort with previously treated advanced NSCLC, nivolumab significantly improved OS compared with docetaxel, with a median OS of 12.0 versus 9.6 months (HR, 0.68; *P* = 0.0006). Nivolumab also achieved a higher ORR (17% vs. 4%) and more durable responses (median duration of response [DOR] not reached vs. 5.3 months), while demonstrating a more favorable safety profile with fewer grade ≥3 treatment-related AEs (10% vs. 48%). These findings were consistent with the global CheckMate 017 (NCT01642004, squamous) and 057 (NCT01673867, nonsquamous) studies in advanced NSCLC, supporting the efficacy and applicability of PD-1 inhibition in Asian patients with advanced NSCLC (50, 51).

Given evidence that pembrolizumab doses above 2 mg/kg provide no added benefit, along with lower body weight and cost constraints in Asian settings, reduced-dose strategies have been explored. A Singapore study in advanced NSCLC found that pembrolizumab 100 mg every 3 weeks achieved comparable outcomes to 200 mg, with similar PFS (6.8 vs 4.2 months), OS (14.3 vs 19.8 months), and response rates (28.6% vs 23.4%). Compared with the standard regimen, the 100-mg dose reduced treatment costs by approximately USD 30, 800 per patient, with an estimated total within-study cost saving of about USD 3.30 million, while maintaining comparable efficacy (52). These cost reductions may improve affordability and patient access and enhance the feasibility of initiating future clinical trials. Similarly, a Malaysian retrospective study of low-dose nivolumab (40 mg every 2 weeks) in six patients with advanced NSCLC showed a mean PFS of 18.4 weeks with minimal toxicity, though conclusions are limited by very small sample size (53).

NPC represents a key disease setting for PD-1 immunotherapy development in Asia due to its higher prevalence, particularly in East and Southeast Asian populations. In a phase I/II study of Chinese patients with NPC and other solid tumors (NCT02593786.), nivolumab demonstrated a manageable safety profile, with treatment-related AEs occurring in 76% of patients, predominantly grade 1–2, and no new safety signals identified. Pharmacokinetic analyses showed comparable nivolumab exposure between Chinese and global populations, suggesting no clinically meaningful ethnic differences in drug disposition. Preliminary anti-tumor activity was observed, with objective responses in 6/46 (13%) evaluable patients, including 4/32 (13%) patients with NPC (median follow-up, 7.5 months; median PFS, 3.5 months), supporting the potential role of PD-1 blockade in advanced NPC (54).

Beyond monotherapy, combination strategies are being actively explored to overcome resistance and enhance PD-1 efficacy. In patients with PD-L1-high (≥50%) metastatic NSCLC, the addition of the IDO1 inhibitor epacadostat to pembrolizumab did not improve outcomes compared with pembrolizumab alone, with similar ORR (32.5% vs. 39.0%), median PFS (6.7 vs. 6.2 months), and no improvement in OS (NCT03322540). The combination was generally well tolerated, with low rates of serious AEs, including pneumonia (4.0%) and pneumonitis (2.7%). However, incomplete normalization of circulating kynurenine levels suggested inadequate IDO1 pathway inhibition, which may have contributed to the lack of clinical benefit (55).

PD-1-based chemoimmunotherapy has also demonstrated benefit in Asian populations. In the China subgroup of the phase III KEYNOTE-966 trial (NCT04003636), first-line pembrolizumab plus gemcitabine and cisplatin for advanced biliary tract cancer (BTC) showed consistent efficacy and safety with the global population. Compared with chemotherapy alone, pembrolizumab plus chemotherapy improved median OS (14.1 vs. 9.9 months; HR, 0.74), ORR (36.0% vs. 28.9%), and DOR (10.2 vs. 5.7 months), with comparable grade 3–4 treatment-related AEs (71.6% vs. 70.7%) and no new safety concerns. Outcomes in the Chinese subgroup were numerically favorable compared with the global cohort, including longer OS (14.1 vs. 12.7 months), higher ORR (36.0% vs. 28.7%), and longer DOR (10.2 vs. 9.7 months) (56, 57).

In Malaysia, the ongoing PIPER trial (NCT05286619) is a novel phase II multicentre, single-arm study evaluating first-line pembrolizumab plus platinum–gemcitabine in recurrent/metastatic HNSCC; no clinical outcomes are yet reported. Its novelty lies in a Simon two-stage design incorporating exploratory biomarker and microbiome analyses to address regional gaps in treatment response and patient stratification (58).

Despite these advances, challenges remain, including heterogeneous responses, immune-related AEs, and limited access in resource-constrained settings like Southeast Asia due to high costs and inadequate biomarker infrastructure (59). Ongoing trials across Asia are evaluating novel PD-1-based combinations and next-generation immunotherapies, highlighting the region’s expanding role in immuno-oncology development.

Ongoing clinical trials across China, Japan, South Korea, India, Taiwan, Singapore, Malaysia, Philippines and Vietnam are evaluating PD-1-based approaches across numerous cancers including NSCLC, gastric cancer, HCC, NPC, esophageal squamous cell carcinoma (ESCC), bladder, cancer, colorectal cancer, cervical cancer, breast cancer, CRC, BTC, RCC and hematologic malignancies. rial activity varies considerably across the region, with China leading by a substantial margin, followed by Japan and South Korea, whereas India and Vietnam currently contribute relatively fewer studies. These investigations encompass a wide range of emerging approaches, including bispecific antibodies targeting PD-1/LAG-3, PD-1/TIGIT, and PD-1/HER2; combinations of PD-1 inhibitors with antibody-drug conjugates (ADC), such as Trop-2- and Nectin-4-targeting agents; targeted therapies (e.g., HIF-2α inhibitors, tyrosine kinase inhibitors, and KRAS inhibitors), oncolytic viruses, therapeutic cancer vaccines, acupuncture, and other immune-modulating interventions (**Supplementary Table 1**). Collectively, these studies underscore Asia’s expanding role in the development of next-generation PD-1–based immunotherapies tailored to regional cancer epidemiology, tumor biology, and unmet clinical needs.

#### 3.1.2 PD-L1

PD-L1 (Programmed Death-Ligand 1) is an immune checkpoint ligand expressed on tumor and immune cells that binds PD-1 on T cells, suppressing T-cell activation and promoting immune evasion. Its surface expression is regulated by post-translational modifications, such as N-glycosylation, as well as internalization, recycling, and degradation, which influence PD-L1–PD-1 interactions and responses to PD-1/PD-L1 inhibitors (38, 60). PD-L1 inhibitors, including atezolizumab and durvalumab, are FDA-approved for NSCLC, urothelial carcinoma, and triple-negative breast cancer (TNBC), with ORRs of 15–30% (61).

In global phase III trials, PD-L1-based immunotherapy has shown benefit in both extensive-stage small cell lung cancer (SCLC) and unresectable stage III NSCLC. In extensive-stage SCLC, IMpower133 (NCT02763579) demonstrated improved OS with atezolizumab plus carboplatin–etoposide versus chemotherapy alone (median OS 12.3 vs. 10.3 months; HR 0.76), with benefit independent of PD-L1 or blood-based tumor mutational burden (bTMB) status and rare reports of durable survival beyond 6 years (62–64). In unresectable stage III NSCLC, PACIFIC-5 (NCT03706690) showed that consolidation durvalumab improved PFS after chemoradiotherapy (median PFS 14.0 vs. 6.5 months; HR 0.75) with a trend toward improved OS (HR 0.87) (65). In previously treated urothelial/urinary tract carcinoma, the phase 3b STRONG (NCT03084471) study showed that durvalumab had a manageable safety profile with immune-related AEs consistent with prior studies and achieved an ORR of 18% with a median OS of 7.0 months, consistent with established activity of PD-L1 blockade in this setting (66). In the phase III NIAGARA trial (NCT03732677), perioperative durvalumab plus chemotherapy improved event-free survival and overall survival versus chemotherapy alone in muscle-invasive bladder cancer (24-month EFS 67.8% vs. 59.8%; 24-month OS 82.2% vs. 75.2%) with comparable grade 3–4 toxicity (67).

In contrast, in advanced gastric or gastroesophageal junction (GEJ) cancer, the phase III JAVELIN Gastric 100 (NCT02625610) trial showed that avelumab maintenance after first-line chemotherapy did not improve OS versus continued chemotherapy in either the overall population (median OS 10.4 vs. 10.9 months; HR 0.91) or the prespecified PD-L1-positive subgroup, although exploratory analyses suggested a possible benefit in patients with PD-L1-positive (combined positive score ≥1); avelumab was also associated with fewer grade ≥3 treatment-related AEs (12.8% vs. 32.8%) (68). Similarly, the phase III JAVELIN Gastric 300 (NCT02625623) trial demonstrated no improvement in OS, PFS, or ORR with third-line avelumab versus physician’s choice chemotherapy, despite a more favorable safety profile (69).

In the Japanese extensive stage SCLC subgroup of IMpower133, atezolizumab plus carboplatin–etoposide showed consistent efficacy versus the global population, with improved OS (14.6 vs. 11.9 months; HR 0.72) and PFS (4.5 vs. 4.0 months; HR 0.47), alongside a manageable safety profile and no treatment-related deaths (70).

In Chinese patients with metastatic NSCLC, the exploratory cohort of the phase III NEPTUNE trial (NCT02542293) showed that first-line durvalumab plus tremelimumab demonstrated a trend toward improved OS compared with chemotherapy, particularly in patients with PD-L1 tumor cell (TC) expression <1% (median OS, 15.0 vs. 11.7 months; HR 0.60, 95% CI 0.32–1.11), although PFS was similar between treatment arms. The combination demonstrated a manageable safety profile, with lower rates of grade 3/4 treatment-related AEs (31.2% vs. 52.6%) and treatment discontinuations due to treatment-related AEs (3.9% vs. 10.3%) than chemotherapy. In contrast, the global phase III NEPTUNE population showed no OS advantage with first-line durvalumab plus tremelimumab over chemotherapy in either the PD-L1 TC <1% subgroup or the intention-to-treat population (ITT median OS 10.9 vs. 12.1 months), although the safety profile was consistent with previous reports and no new safety signals were observed (71). Similarly, Similarly, in the phase III MYSTIC trial (NCT02453282), neither durvalumab monotherapy nor durvalumab plus tremelimumab improved OSs versus chemotherapy in PD-L1-selected metastatic NSCLC, although exploratory analyses suggested a potential OS benefit of the combination in patients with high bTMB ≥20 mut/Mb (21.9 vs. 10.0 months; HR 0.49) (72).

Multiple clinical trials across Asian populations are evaluating PD-L1-based therapies across a broad range of malignancies, including BTC, HCC, cervical cancer, HNSCC, ESCC, NSCLC, and SCLC. China accounts for the largest proportion of studies, with NSCLC representing the most extensively investigated indication. The main therapeutic strategies involve combination approaches with chemotherapy and anti-CTLA-4 agents, alongside other combinations such as anti-EGFR and anti-VEGF therapies. Emerging investigations further include bispecific antibodies targeting PD-L1/VEGF, PD-L1/4-1BB, and PD-1/LAG-3, as well as PD-L1-based combinations with ADCs (e.g., B7-H3 and Nectin-4), targeted therapies (including tyrosine kinase and KRAS inhibitors), and novel immunotherapeutic platforms such as microbiome-modulating strategies and DLL3-directed bispecific T-cell engagers (**Supplementary Table 1**). However, in Malaysia, limited access to PD-L1 testing contributes to a lower number of clinical trials and hampers optimal patient selection. This reflects broader disparities in biomarker-driven trial implementation across the region, which may influence patient enrollment and study distribution (48, 49, 59, 73–76).

#### 3.1.3 CTLA-4

CTLA-4 (Cytotoxic T-Lymphocyte-Associated Protein 4) is an immune checkpoint that regulates early T-cell activation by competing with CD28 for CD80/CD86 binding on antigen-presenting cells, thereby inhibiting costimulatory signaling. Upon activation, CTLA-4 translocates to the T-cell surface and recruits inhibitory molecules, reinforcing immune suppression and tolerance (38). Antibodies such as ipilimumab and tremelimumab restore T-cell activation and anti-tumor immunity; ipilimumab, approved in 2011 for advanced melanoma, marked a milestone in immunotherapy (77), and is now used in combination regimens for cancers including renal cell carcinoma (RCC) and HCC (78, 79). However, CTLA-4 blockade is associated with higher immune-related AEs and modest monotherapy response rates (~10–25%) (14, 80).

As shown in Section 3.1.2 (PD-L1), CTLA-4-based combination immunotherapy has demonstrated heterogeneous clinical activity across malignancies, including a numerical OS benefit with tremelimumab plus durvalumab in the Chinese exploratory NEPTUNE phase III trial (NCT02542293) in PD-L1-low metastatic NSCLC (71), and a similar numerical OS benefit in the global ARCTIC phase III trial (NCT02352948) in patients with PD-L1 <25%, with both studies showing limited impact on PFS (81). In the Asian subpopulation of CheckMate 227 (NCT02477826; Japan, South Korea, Taiwan), nivolumab plus ipilimumab provided durable survival benefit versus chemotherapy (3-year OS 53% vs 37%; HR 0.72) (82), while in EBV-positive recurrent/metastatic NPC (NCT03097939), dual CTLA-4/PD-1 blockade achieved a 38% response rate with median OS of 19.5 months and manageable toxicity (83). Collectively, these findings suggest that CTLA-4-based combinations confer benefit in selected biomarker-defined and virus-associated subgroups, highlighting the need for further stratification of predictors of response and inter-population variability.CTLA-4 blockade (ipilimumab), alone or in combination with PD-1 inhibitors, has been associated with rare but potentially life-threatening immune-mediated cardiotoxicities, including myocarditis, cardiomyopathy, heart failure, cardiac fibrosis, and cardiac arrest. In a multicenter case series, 5 of 8 patients (63%) with cardiotoxicity also developed multisystem immune-related AEs (84), while a meta-analysis of 20 randomized trials demonstrated increased risks of all-grade cardiovascular toxicity (OR 1.33, 95% CI 1.00–1.75) and severe cardiovascular AEs with CTLA-4 inhibitor monotherapy (OR 2.00, 95% CI 1.08–3.70) (85). Overall, CTLA-4 inhibitors are associated with a higher incidence of immune-related AEs than PD-1/PD-L1 inhibitors, with combined CTLA-4/PD-1 blockade conferring the greatest toxicity burden.

In Asia, CTLA-4-based clinical development is predominantly focused on combination strategies rather than single-agent therapy, particularly next-generation immunotherapy platforms. These include bispecific antibodies (e.g., PD-1/CTLA-4 and PD-L1/CTLA-4 constructs), dual-affinity re-targeting (DART) molecules targeting PD-1/CTLA-4, and emerging trispecific antibodies (e.g., PD-1/CTLA-4/VEGF-A), alongside combinations with other ICIs (e.g., LAG-3, CD38), chemotherapy, tyrosine kinase inhibitors, HIF-2α and PARP inhibitors, and ADCs (e.g., Trop-2, Claudin18.2, nectin-4 AND VEGF). These CTLA-4-based strategies are being evaluated across multiple tumor types, with NSCLC and HNSCC as the leading indications, followed by SCLC, NPC, BTC, gastric, colorectal, bladder, HCC, RCC, glioblastoma, and virus-associated malignancies. Regionally, China leads CTLA-4-based development, followed by South Korea, Taiwan, and Japan, while activity in Southeast Asia remains limited, with only isolated studies (e.g., a single NSCLC trial reported in Malaysia) (**Supplementary Table 1**).

#### 3.1.4 TIM-3

TIM-3 (T cell immunoglobulin and mucin-domain containing-3) is an inhibitory checkpoint receptor expressed on exhausted T cells and innate immune cells, often co-expressed with PD-1 in TME, where it promotes immune tolerance and suppresses anti-tumor immunity. Through interactions with ligands such as Galectin-9 and CEACAM1, TIM-3 inhibits T-cell proliferation and cytokine production while enhancing immunosuppressive cells like myeloid-derived suppressor cells (MDSCs), and its expression correlates with poor prognosis in cancers including NSCLC, cervical, and gastric cancer (86–88).

Preclinical studies showing enhanced anti-tumor effects of TIM-3 blockade, particularly with PD-1 inhibition, have driven clinical trials in myelodysplastic syndrome (MDS), acute myeloid leukemia (AML), and solid tumors. Sabatolimab, with FDA Fast Track and European Union orphan designation for MDS, achieved ORRs of 50% in high-risk and 85% in very high-risk patients when combined with hypomethylating agents. Cobolimab demonstrated a 42.9% ORRs with dostarlimab in NSCLC and is advancing in phase 2/3 trials in numerous cancers (e.g. lung, liver and melanoma) (87, 88). TIM-3 blockade is mainly explored in combination regimens due to modest monotherapy efficacy. Since 2016, at least eight anti-TIM-3 or PD-1/TIM-3 bispecific antibodies have entered clinical trials, including TSR022 and MBG453, which achieved a 40% ORR in AML patients (89), demonstrating manageable safety and promising early efficacy; however, optimal design and TIM-3 biology remain under investigation (87, 88).

In a first-in-human phase I study of the PD-1/TIM-3 bispecific antibody lomvastomig (NCT03708328; multicenter, international trial), patients with advanced solid tumors included ICI-experienced melanoma and NSCLC and ICI-naïve SCLC and ESCC. The agent demonstrated a manageable safety profile, with one dose-limiting toxicity (grade 3 troponin T elevation) and no maximum tolerated dose reached up to 2, 100 mg every 2 weeks. Objective responses were observed in 21% of patients in dose escalation, 8% in ICI-experienced melanoma, and 20% in ICI-naïve ESCC, with limited activity in ICI-experienced NSCLC but more encouraging efficacy in ICI-naïve ESCC, supporting greater TIM-3 pathway relevance in less pretreated disease (90).

In Asia, TIM-3-based immunotherapy development remains less extensive than PD-1, PD-L1, and CTLA-4 pathways, but is expanding across a range of malignancies including NSCLC, ESCC, urothelial carcinoma, NPC, melanoma, colorectal cancer, HNSCC, lymphoma, MDS, and AML. Development activity is concentrated in China and South Korea, followed by Taiwan, while most other Asian countries conduct only one to two early-phase studies with limited indication coverage; examples include NSCLC in Hong Kong, MDS/AML in Malaysia, and ESCC in Singapore and Thailand. Therapeutic strategies are predominantly combination-based, including PD-1/TIM-3 bispecific antibodies and regimens combining TIM-3 inhibition with PD-1/PD-L1 blockade, CD73, NKG2A, TIGIT, CD47, and LAG-3 inhibitors, as well as targeted agents such as BCL2, PARP, demethylating agents, tyrosine kinase inhibitors, IL-6 inhibitors, and ADCs (e.g., nectin-4, Trop-2), alongside emerging bispecific and multi-target platforms such as Claudin18.2/IL-10 and PD-1/LAG-3, reflecting increasing diversification of TIM-3-targeted strategies across both hematologic and solid tumors (**Supplementary Table 1**).

#### 3.1.5 LAG-3

LAG-3 (Lymphocyte Activation Gene-3) is an inhibitory checkpoint receptor that suppresses T-cell activity by binding major histocompatibility complex (MHC) class II and interacting with the T-cell receptor (TCR)-CD3 complex, often co-expressed with PD-1 on exhausted T cells. Its function is modulated by proteolytic cleavage via ADAM10/17, affecting anti-tumor immunity and response to PD-1 blockade (87, 88).

LAG-3 blockade has reached clinical application, with FDA approval of nivolumab plus relatlimab (Opdualag) in 2022 for unresectable or metastatic melanoma. In the global phase II–III RELATIVITY-047 trial (NCT03470922), previously untreated metastatic or unresectable melanoma patients received either relatlimab plus nivolumab or nivolumab alone, with the combination significantly improving median PFS (10.1 vs 4.6 months; HR 0.75) and 12-month PFS rates (47.7% vs 36.0%). Grade 3–4 treatment-related AEs were more frequent with combination therapy (18.9% vs 9.7%), although no new safety signals were observed, indicating a manageable safety profile in this global population (91). Early studies of the LAG-3 fusion protein IMP321 (eftilagimod alpha; NCT00351949, NCT00349934) also demonstrated encouraging activity with manageable toxicity, achieving stable disease in 7/8 patients with metastatic RCC and improving ORRs in HER2-negative metastatic breast cancer when combined with paclitaxel (50% vs. 25%) (88). In contrast, in the phase II RELATIVITY-060 trial (NCT03662659) in previously untreated unresectable or metastatic gastric or GEJ cancer, addition of relatlimab to nivolumab plus chemotherapy failed to improve efficacy in LAG-3-positive tumors (ORR 48% vs 61%; median PFS 7.0 vs 8.3 months; HR 1.41; median OS 13.5 vs 16.0 months; HR 1.04) and was associated with higher grade 3–4 AEs (69% vs 61%) and treatment discontinuation (42% vs 36%), indicating increased toxicity without clinical benefit in this global gastric or GEJ cancer patients (92).

Recent LAG-3-targeted development in Asia is expanding, despite mixed clinical outcomes across tumor types. China remains the leading contributor to clinical research, followed by South Korea and Taiwan, with additional early-phase activity in Japan, Singapore, Malaysia, Thailand, and India. Development is mainly focused on Asian-prevalent malignancies, including NSCLC, HCC, HNSCC, NPC, ESCC, colorectal cancer, urothelial carcinoma, gastric cancer, virus-associated tumors, also including blood malignancies such as Hodgkin lymphoma, and diffuse large B-cell lymphoma (DLBCL). Therapeutic strategies are predominantly combination-based, including PD-1/LAG-3 bispecific antibodies and combinations with PD-1, CTLA-4, and TIGIT blockade, alongside targeted agents (HER2, CD38, CD47), inhibitors (VEGF, tyrosine kinase, IDO), and chemotherapy (**Supplementary Table 1**), reflecting a broad but clinically heterogeneous and still evolving development landscape across the region.

#### 3.1.6 TIGIT

TIGIT (T cell immunoreceptor with Ig and ITIM domains) is an inhibitory checkpoint expressed on T cells, regulatory T cells (Tregs), and NK cells, regulating immune responses by interacting with ligands CD155 and CD112 and competing with the co-stimulatory receptor CD226. It is upregulated in multiple cancers, including NSCLC, melanoma, breast, colon, gastric, AML, and multiple myeloma (87, 88, 93–95).

In the first-in-human phase I dose-escalation study (NCT04047862), ociperlimab (anti-TIGIT) plus tislelizumab (anti-PD-1) showed modest activity in previously treated advanced solid tumors (ORR 10%, disease control rate (DCR) 50%, median DOR 3.6 months), with no dose-limiting toxicities or maximum tolerated dose identified. Despite frequent grade ≥3 AEs (62.5%), the combination demonstrated a manageable safety profile and advanced to phase II evaluation (96). Similarly, the phase I first-in-human study of M6223 (anti-TIGIT; NCT04457778), administered alone or with bintrafusp alfa (TGF-β/PD-L1 inhibitor), demonstrated a manageable safety profile with no maximum tolerated dose identified in advanced solid tumors. Clinical activity was limited, with stable disease as the best response in approximately one-third of patients, supporting further evaluation of TIGIT-based combination strategies (97).

Phase II studies have shown encouraging but heterogeneous activity for TIGIT blockade across tumor types. In recurrent/metastatic cervical cancer (NCT04693234), ociperlimab plus tislelizumab achieved an ORR of 23.2% (27.4% in PD-L1-positive tumors) and a median OS of 12.2 months (16.4 months in PD-L1-positive patients), with 18.1% grade ≥3 treatment-related AEs (98). In advanced ESCC (AdvanTIG-203; NCT04732494), the combination improved ORR versus tislelizumab alone (30.6% vs. 20.6%) but did not improve PFS (HR 0.93) or OS (HR 0.93), with similar rates of grade ≥3 AEs (~41% in both arms) (99). Similarly, in limited-stage SCLC (AdvanTIG-204; NCT04952597), adding ociperlimab to tislelizumab plus concurrent chemoradiotherapy did not improve PFS (12.6 vs. 13.2 months; HR 0.84), with frequent but manageable grade ≥3 treatment-related AEs (73.2% vs. 78.6% vs. 65.1% across arms) (100). In contrast, the phase II CITYSCAPE trial (NCT03563716) demonstrated improved ORR (31.3% vs. 16.2%) and PFS (5.4 vs. 3.6 months; HR 0.57) with tiragolumab (anti-TIGIT) plus atezolizumab (anti-PD-L1) in PD-L1-positive metastatic NSCLC, with grade ≥3 AEs more common in the combination arm (e.g., lipase elevation 9% vs. 3%) and two treatment-related deaths reported (101). Translational analyses further suggested that benefit was associated with tumors enriched for intratumoral macrophages and regulatory T cells, supporting a potential biomarker-defined population most likely to benefit from TIGIT blockade (102).

In a broader multicohort across the KEYVIBE program (KEYVIBE-001/002/002C), vibostolimab (anti-TIGIT) plus pembrolizumab showed modest, heterogeneous activity across tumor types, with ORRs of ~15–23% in cervical cancer, ~24% in PD-L1–positive ovarian cancer, and ~27% in gastric cancer. In KEYVIBE-002 (metastatic NSCLC), it did not significantly improve PFS versus docetaxel (HR 0.77), while KEYMAKER-U02C (melanoma) reported a 38% pathologic complete response rate in the neoadjuvant setting. Overall, efficacy was inconsistent across indications, with a safety profile generally consistent with PD-1-based therapy (103).

Despite encouraging early-phase signals, phase III trials have not shown meaningful clinical benefit from TIGIT inhibition. In SKYSCRAPER-02 (NCT04256421; ES-SCLC), tiragolumab plus atezolizumab and chemotherapy showed no improvement in PFS (5.4 vs 5.6 months; HR 1.11) or OS (13.1 vs 13.1 months; HR 1.14), with similar rates of grade 3/4 immune-related AEs (~8%) and discontinuation (~8–9%) (104). In SKYSCRAPER-01 (NCT04294810; PD-L1-high NSCLC), the combination improved PFS (7.0 vs 5.6 months; HR 0.78) and ORR (45.8% vs 35.1%) but not OS (23.1 vs 16.9 months; HR 0.87), while increasing grade 3–4 AEs (41.2% vs 33.8%), treatment withdrawal (16.1% vs 6.5%), and grade 5 events (10.9% vs 9.9%) (105). In adjuvant melanoma (NCT05665595), vibostolimab plus pembrolizumab failed to improve recurrence-free survival (HR 1.25) and led to early termination for futility, with higher rates of serious immune-related toxicities, including rare fatal events (106).

Collectively, clinical experience with anti-TIGIT antibodies, including ociperlimab, tiragolumab, vibostolimab, and M6223, demonstrates that TIGIT blockade is generally feasible and has a manageable safety profile when combined with PD-1/PD-L1 inhibitors. Although early-phase studies showed encouraging activity in selected settings, particularly cervical cancer and PD-L1-positive NSCLC, these findings have not translated into consistent improvements in PFS or OS in randomized phase III trials. Overall, the clinical benefit of TIGIT blockade appears limited and context-dependent, with emerging translational data suggesting that efficacy depends on tumor biology and the immune microenvironment, emphasizing the need for selection of patients most likely to benefit.

Among emerging ICI targets, TIGIT is one of the most extensively investigated, with broad clinical development across multiple tumor types and geographies. Key indications include NSCLC (dominant), HNSCC, urothelial carcinoma, BTC, cervical cancer, glioma, gastric cancer, HCC, hepatobiliary tumors, endometrial cancer, melanoma, and other advanced solid tumors. Geographically, clinical development is most active in Asia, led by China and South Korea with the highest level of innovation and combination strategies. This is followed by Japan and Taiwan, then Thailand, India, Malaysia, and Hong Kong (typically 1–2 indications), while the Philippines and Vietnam are largely limited to NSCLC studies. Therapeutic strategies include both monotherapy and extensive combination approaches. These range from bispecific antibodies (e.g., PD-1/TIGIT such as rilvegostomig and BC008-1a), TIGIT/PVRIG, PD-L1/TIGIT, and TIGIT/TGF-β bispecific antibodies, to co-formulations (e.g., MK-7684A). Combination agents span ICIs (PD-1, PD-L1, CTLA-4, CD47), targeted therapies (VEGF/VEGFR2, HER2/topoisomerase I, PARP1, adenosine receptors A2A/A2B, ICOS, CD96, CD73, IL-15R, IL-6), ADCs (e.g., B7-H4, Trop-2, Claudin18.2), other bispecific antibody combinations (PD-1/LAG-3, PD-1/CTLA-4), chemotherapy, and emerging immunomodulator such as CD8-targeted IL-2 (**Supplementary Table 1**).

#### 3.1.7 VISTA

VISTA (V-domain Ig Suppressor of T Cell Activation) is a type I transmembrane checkpoint receptor related to PD-1/PD-L1, with a single IgV-like domain. Highly expressed on myeloid cells and at lower levels on T cells, VISTA contributes to immune homeostasis (107, 108). In tumors, VISTA is often upregulated and drives PD-1 resistance by binding LRIG1, inducing quiescence in tumor-specific CD8^+^ T cells (109). VISTA, acting as both ligand and receptor, suppresses T cells via myeloid and Treg cells, and blockade enhances responses, especially after PD-1 or CTLA-4 inhibition (108). VISTA is expressed in pancreatic, melanoma, NSCLC, CRC, and breast cancers, with high levels linked to poor immunotherapy outcomes, supporting co-targeting strategies (110).

VISTA-targeting antibodies are emerging immune checkpoint inhibitors designed to reverse myeloid- and T-cell-mediated immunosuppression, with three leading agents currently in early clinical development: CI-8993 (Curis, Inc.), HMBD-002 (Hummingbird Bioscience), and KVA12123 (Kineta, Inc.). These agents differ in antibody design and clinical strategy but collectively aim to modulate the VISTA pathway and enhance responses to existing immunotherapies, particularly PD-1 blockade. CI-8993 (Curis, Inc.; NCT04475523) (111, 112), HMBD-002 (Hummingbird Bioscience; NCT05082610) (111, 113)., and KVA12123 (Kineta, Inc.; NCT05708950) (111, 114) are all being evaluated in early-phase clinical trials in patients with advanced solid tumors, reflecting growing clinical interest in VISTA as a next-generation immuno-oncology target. Additional investigational agents include BMS-767 (Bristol Myers Squibb) and W0180 (Pierre Fabre), further underscoring the expanding pipeline of VISTA-directed therapeutics.

CI-8993 (Curis, Inc.; NCT04475523) is a first-in-class anti-VISTA IgG1 antibody evaluated in a phase I dose-escalation study in relapsed/refractory metastatic or unresectable solid tumors. It showed pharmacodynamic activity at 0.15 mg/kg with immune activation (increased T cells and dendritic cells, reduced MDSCs) and preclinical tumor growth inhibition. Safety was manageable, with no dose-limiting toxicities at low doses, though transient cytokine release syndrome was reported (112).

KVA12123 is a fully human anti-VISTA monoclonal antibody developed to enhance anti-tumor immunity by blocking the VISTA immune checkpoint. Preclinical studies showed high specificity, immune activation, and strong anti-tumor efficacy in mouse models, with improved activity in combination with anti-PD-1 therapy. In cynomolgus monkeys, it demonstrated a favorable safety profile with no significant toxicity or cytokine release signals. The antibody has progressed into a Phase 1/2 clinical trial in patients with advanced solid tumors, evaluating monotherapy and combination with pembrolizumab (115).

SNS-101 is a pH-selective anti-VISTA monoclonal antibody designed to overcome limitations of earlier VISTA-targeting agents, particularly rapid systemic clearance and cytokine release syndrome (CRS), while improving pharmacokinetics, safety, and responsiveness to PD-1 blockade. In human VISTA knock-in syngeneic tumor models, SNS-101 showed significant anti-tumor efficacy only in combination with PD-1 inhibition, accompanied by modulation of cytokine and chemokine signaling and remodeling of the TME. In contrast to earlier anti-VISTA antibodies associated with CRS and unfavorable pharmacokinetics, SNS-101 exhibited improved pharmacokinetic stability and no reported CRS signals in preclinical assessments, consistent with its pH-dependent binding and distinct epitope targeting. Overall, preclinical toxicity evaluations revealed no dose-limiting or clinically relevant immune-related safety concerns, supporting a broader therapeutic window compared with first-generation VISTA inhibitors while retaining immune-activating anti-tumor activity (116).

Compared with established ICIs, clinical development of VISTA-targeting strategies remains very limited. The only reported clinical study is the small-molecule CA-170 (Curis, Inc.; NCT02812875), an oral dual PD-L1/PD-L2 and VISTA inhibitor evaluated in early-phase trials in patients with advanced solid tumors and lymphomas, with South Korea representing the only Asian study site (**Supplementary Table 1**).

### 3.2 Targeted antibodies

Targeted antibody therapies have revolutionized modern oncology by enabling the precise recognition and elimination of cancer cells through tumor-associated antigens, thereby minimizing damage to healthy tissues and improving therapeutic efficacy in selected patient populations. The two main classes dominating this space: monoclonal antibodies (mAbs) and bispecific antibodies (bsAbs), which differ in their mechanisms of action, biological targets, clinical performance, and regulatory status. In Asia and Southeast Asia, the development and adoption of targeted antibody therapies have accelerated, driven by expanding participation in regional clinical trials and growing investment in oncology research.

#### 3.2.1 Monoclonal antibodies

mAbs are engineered to recognize a single, specific antigen, often a tumor surface receptor, inhibiting tumor growth through receptor blockade or immune-mediated cytotoxicity. By binding tumor cells, mAbs can disrupt critical signaling pathways and recruit immune effector cells for targeted elimination. The first FDA-approved mAb, rituximab (1997), targets CD20 on B cells for the treatment of lymphoma (117) and has since been widely approved and adopted across Asia and Southeast Asia, including Singapore (118) and Malaysia (119). Since then, numerous mAbs have been approved for diverse malignancies, including ICIs such as pembrolizumab and nivolumab (PD-1), ipilimumab (CTLA-4), and atezolizumab (PD-L1). Other notable agents include trastuzumab and pertuzumab (HER2) for breast cancer; bevacizumab (VEGF) and cetuzimab and panitumumab (EGFR) for CRC; necitumumab (EGFR) for NSCLC; ramucirumab (VEGFR2) for gastric cancer; brentuximab vedotin (CD30) for hodgkin lymphoma; and dinutuximab (GD2) for neuroblasto (120).

In addition, numerous mAbs, including biosimilars and novel agents, have been developed in Asia, particularly in China. Among these, camrelizumab demonstrated an ORR of 14.7% and a 6-month overall survival rate of 74.4% (121). Similarly, toripalimab, a humanized anti-PD-1 mAb, has been approved in China for second-line treatment of melanoma, first- and third-line treatment of NPC, and second-line treatment of urothelial carcinoma, showing promising anti-tumor activity and long-term survival benefits, particularly in Chinese melanoma patients (122). Given the rapid pace of development, the number of approved and investigational mAbs continues to expand, precluding comprehensive coverage in this review.

In Malaysia, biosimilar intravenous trastuzumab has been studied in HER2-positive breast cancer within the public healthcare setting, demonstrating comparable efficacy and safety to the reference product at substantially lower costs due to reduced development expenses. Currently, three biosimilars, Hertraz and Zuhera (registered in 2018) and Herzuma (registered in 2019), are available, increasing market competition and contributing to price reductions (123–125). However, high costs and reimbursement limitations continue to restrict broader access in public healthcare systems. Further development of cost-effective biosimilars tailored to the disease burden in Asia and Southeast Asia remains essential.

#### 3.2.2 Bispecific antibodies

bsAbs represent a rapidly advancing class of targeted antibody therapies, with fifteen agents approved for cancer treatment by 2025. Unlike conventional mAbs, bsAbs are engineered to simultaneously bind two distinct antigens, enabling enhanced specificity and novel mechanisms of action. These include dual pathway inhibition, tumor-targeted agonism, immune cell engagement, and checkpoint or co-stimulatory modulation. Functionally, bsAbs operate through either combinatorial mechanisms, integrating two independent activities within a single molecule, as exemplified by cadonilimab (PD-1 × CTLA-4), or obligate mechanisms requiring coordinated engagement of both targets, as seen in T cell engagers (TCEs) such as blinatumomab, which links CD3 on T cells to CD19 on B cells to induce cytotoxicity (126–128).

In cancer therapy, bsAbs encompass diverse formats, including checkpoint or co-stimulatory modulators, T cell and NK cell engagers, dual-pathway inhibitors, and bispecific antibody–drug conjugates. These agents primarily enhance T cell–mediated anti-tumor immunity through mechanisms such as checkpoint blockade, effector cell recruitment and activation, and co-stimulatory signaling (126–128).

**Table 1** summarizes bsAbs approved by relevant regulatory agencies for oncology indications as of March 2026. Of the fifteen approved bsAbs, twelve are authorized in the United States and/or Europe and two in China (**Table 1**), although catumaxomab was withdrawn in 2013 and 2017, respectively. focusing exclusively on their clinical applications in cancer treatment. Most approved bsAbs are TCEs, with many candidates advancing to phase II–III trials. In Asia, approvals are currently limited to China. Cadonilimab, the first approved bsAb (2022), was initially indicated for relapsed or metastatic cervical cancer refractory to platinum-based chemotherapy (COMPASSION-03) (126, 129–131), and subsequently expanded to first-line treatment of advanced gastric/gastroesophageal junction adenocarcinoma in combination with fluoropyrimidine and platinum-based chemotherapy in 2024 (COMPASSION-15/AK104-302, 2024) (129, 132), as well as persistent, recurrent, or metastatic cervical cancer in combination with platinum-based chemotherapy with or without bevacizumab in 2025 (COMPASSION-16/AK104-303, 2025) (133, 134). Ivonescimab (PD-1 × VEGF) showed superior PFS versus pembrolizumab in the phase III HARMONi-A trial in EGFR-mutant NSCLC, leading to its approval in China in 2025 (135, 136).

**Table 1:** Approved bispecific antibodies.

Name	Targets	Developer	Year of first approval	Indication	Current status
Catumaxomab	CD3 × EpCAM	Trion Pharma	2009 (EMA)	Malignant ascites	Withdrawn
Blinatumomab	CD3 × CD19	Amgen	2014 (FDA), 2015 (EMA)	Acute lymphoblastic leukemia	Active
Amivantamab	EGFR × cMET	Janssen	2021 (FDA)	NSCLC	Active
Mosunetuzumab	CD3 × CD20	Roche	2022 (FDA, EMA)	Relapsed/refractory follicular lymphoma (R/R FL)	Active
Tebentafusp	CD3 × gp100	Immunocore	2022 (FDA, EMA)	Uveal melanoma	Active
Teclistamab	CD3 × BCMA	Janssen	2022 (FDA, EMA)	Relapsed/refractory multiple myeloma (R/R MM)	Active
Cadonilimab	PD-1 × CTLA-4	Akeso	2022 (NMPA)	Recurrent/metastatic cervical cancer (R/M CC)	Active
			2024 (NMPA)	Advanced/metastatic gastric	Active
			2025 (NMPA)	Recurrent/metastatic cervical cancer	Active
Epcoritamab	CD3 × CD20	Genmab	2023 (FDA)	Relapsed/refractory DLBCL and high-grade B-cell lymphoma	Active
Talquetamab	CD3 × GPRC5D	Janssen	2023 (FDA)	Relapsed/refractory multiple myeloma	Active
Elranatamab	CD3 × BCMA	Pfizer	2023 (FDA)	Relapsed/refractory multiple myeloma	Active
Odronextamab	CD3 × CD20	Regeneron	2024 (EMA)	Follicular lymphoma (FL)	Active
Zanidatamab	HER2 × HER2	Jazz Pharma	2024 (FDA)	HER2-positive biliary tract cancer (BTC)	Active
Linvoseltamab	BCMA × CD3	Regeneron	2025 (FDA)	Relapsed/refractory multiple myeloma (R/R MM)	Active
Ivonescimab	PD-1 × VEGF-A	Akeso/Summit	2025 (NMPA)	First-line PD-L1-positive NSCLC	Active
Glofitamab	CD3 × CD20	Roche	2025 (FDA, EMA)	Relapsed/refractory DLBCL	Active

EMA, European Medicines Agency; FDA, US Food and Drug Administration; NMPA, China National Medical Products Administration.

Sources: Klein et al.; Brinkmann et al.; Labrijn et al.; Gao et al.; Chen et al.; Ji et al.; Wu et al.; Dhillon et al.; Akesobio.com; https://clinicaltrials.gov/.

While no bsAbs have yet been approved in Malaysia, the country is participating in regional trials investigating several agents, including odronextamab for non-Hodgkin’s lymphoma (NCT06149286), teclistamab for multiple myeloma (NCT05572515), amivantamab for NSCLC (NCT05801029, NCT05908734, NCT04988295, NCT04487080, NCT04538664, NCT05388669, NCT05498428), CRC (NCT05379595, NCT06750094, NCT06662786), head and neck cancer (NCT06385080), and zanidatamab for gastric and esophageal cancers (NCT05152147). Across Asia, bsAbs are emerging as a versatile class of cancer therapeutics, leveraging dual-target engagement to enhance anti-tumor immunity, inhibit multiple signaling pathways, and expand treatment options for both hematologic and solid malignancies.

#### 3.2.3 Antibody-drug conjugates

Antibody-drug conjugates (ADCs) entered clinical oncology with the first FDA approval of gemtuzumab ozogamicin (Mylotarg) in 2000 for acute myeloid leukemia, marking the beginning of this therapeutic class. As of August 2024, 378 ADCs had been developed globally, including 11 FDA-approved agents, 217 in clinical development, and 150 discontinued; by 2026, the number of approved ADCs had increased to 15 (**Table 2**), spanning both hematologic malignancies and solid tumors. Early-generation ADCs were limited by modest efficacy and substantial toxicities, including hematologic, hepatic, gastrointestinal, ocular, and pulmonary AEs, which contributed to the discontinuation or withdrawal of several agents. For example, gemtuzumab ozogamicin was temporarily withdrawn before being re-approved with an optimized dosing schedule, while moxetumomab pasudotox (Lumoxiti, AstraZeneca) was permanently withdrawn from the U.S. market in 2023 because of limited clinical uptake rather than safety or efficacy concerns. In contrast, contemporary ADCs have demonstrated improved clinical outcomes, including higher response rates and prolonged PFS compared with standard chemotherapy in multiple trials, leading to increased regulatory approvals over the past decade. Despite these advances, toxicity and variable efficacy remain key limitations that continue to shape dosing strategies, patient selection, and combination approaches (137–139).

**Table 2:** Geographic distribution of ongoing Antibody-Drug Conjugate (ADC) clinical trial sites across Asian countries.

ADC target	Indication	Trial sites (Asian Countries)	NCT number
B7-H3	SCLC	China, South Korea	NCT06898957
B7-H4	Advanced solid tumors	China, Japan, South Korea, Taiwan, Thailand	NCT05123482
Claudin 18.2	Gastric/GEJ adenocarcinoma	China, Japan, South Korea, Taiwan	NCT05702229,
	Advanced solid tumors	China	NCT06468358
HER3	NSCLC	China	NCT07111520
PD-L1	NSCLC	China	NCT06907615, NCT04316364
	Cervical or Ovarian cancer	China	NCT06769152
	ESCC	China	NCT06769113
	HCC	China	NCT06742892
	HNSCC	China	NCT06857279
	Advanced solid tumors	China	NCT06848699
Nectin-4	Bladder cancer	Hong Kong, Japan, South Korea, Taiwan, Thailand, Vietnam	NCT04960709
	MIBC	China, Japan, Malaysia, Philippines, Singapore, South Korea, Taiwan	NCT04700124
	Urothelial carcinoma	South Korea, Taiwan	NCT03869190
Trop-2	NSCLC	China, Japan, Singapore, South Korea, Malaysia, Taiwan, Thailand	NCT05609968, NCT07098338
	Breast cancer	Hong Kong, India, Japan, Malaysia, Philippines, Singapore, South Korea, Taiwan	NCT06312176
	TNBC	China, Hong Kong, Japan, South Korea, Malaysia, Philippines, Singapore, Taiwan, Thailand, Vietnam	NCT06841354, NCT06966700, NCT05382286
	Urothelial carcinoma	South Korea, Taiwan	NCT03869190, NCT05327530

SCLC, Small cell lung cancer; GEJ, Gastroesophageal Junction; NSCLC, Non-small cell lung cancer; ESCC, Esophageal squamous cell carcinoma; HCC, Hepatocellular carcinoma; HNSCC, Head and neck squamous cell carcinoma; MIBC, Muscle-invasive bladder cancer; TNBC, Triple-negative breast cancer.

Sources: https://clinicaltrials.gov/.

ADC approvals in Asia are concentrated in Japan and China, which together represent the main drivers of regional clinical adoption and innovation. In Japan, approvals are regulated by the Pharmaceuticals and Medical Devices Agency (PMDA), which has led global first approvals for several ADCs, most notably trastuzumab deruxtecan (Enhertu), along with established PMDA approvals for trastuzumab emtansine, brentuximab vedotin, polatuzumab vedotin, and enfortumab vedotin across breast cancer, lymphoma, and urothelial carcinoma. In contrast, China, regulated by the National Medical Products Administration (NMPA), has rapidly expanded domestic ADC development, with approvals including disitamab vedotin (RC48) for HER2-expressing gastric, urothelial, and breast cancers, and emerging NMPA approvals of next-generation agents such as sacituzumab tirumotecan. Other Asian regions, including South Korea (Ministry of Food and Drug Safety, MFDS), Singapore (Health Sciences Authority, HSA), Taiwan (Taiwan Food and Drug Administration, TFDA), Hong Kong, and Malaysia (National Pharmaceutical Regulatory Agency, NPRA), primarily access ADCs through FDA/European Medicines Agency (EMA)-aligned approvals rather than independent development pathways. Overall, Asia represents a major global hub for both first-in-class ADC innovation (Japan/PMDA) and rapid domestic expansion (China/NMPA) (137, 138, 140, 141).

Asia has become a major region for multinational ADC clinical trials, with study sites spanning Asia and Southeast Asia. Trop-2-targeting ADCs have the broadest geographic representation, with trials conducted in China, Hong Kong, India, Japan, South Korea, Malaysia, the Philippines, Singapore, Taiwan, Thailand, and Vietnam across TNBC, breast cancer, NSCLC, and urothelial carcinoma. PD-L1-targeting ADCs are currently evaluated primarily in China for NSCLC, HCC, HNSCC, and ESCC. Other ongoing ADC trials include B7-H3 (China and South Korea; small cell lung cancer), B7-H4 (China, Japan, South Korea, Taiwan, and Thailand; advanced solid tumors), HER3 (China; NSCLC), Claudin 18.2 (China, Japan, South Korea, and Taiwan; gastric and gastroesophageal junction cancers), and Nectin-4 (China, Japan, South Korea, Malaysia, the Philippines, Singapore, Taiwan, Thailand, and Vietnam; muscle-invasive bladder cancer and urothelial carcinoma). Collectively, these studies highlight the growing role of Asian countries as major recruitment hubs for multinational ADC clinical trials across a broad spectrum of solid tumors (**Table 3**).

**Table 3:** FDA-approved of Antibody–Drug Conjugate (ADC).

Drug	Brand name	Company	Target	1st approval (Year); Status	Indication
Gemtuzumab ozogamicin	Mylotarg	Pfizer	CD33	2000; Voluntarily withdrawn in 2010; Reapproved in 2017	AML
Brentuximab vedotin	Adcetris	Seagen (now Pfizer)/Takeda	CD30	2011	HL, ALCL
ad0-Trastuzumab emtansine	Kadcyla	Genentech/Roche	HER2	2013	HER2+ Breast Cancer
Inotuzumab ozogamicin	Besponsa	Wyeth/Pfizer	CD22	2017	NHL, ALL
Moxetumomab pasudotox	Lumoxiti	AstraZeneca	CD22	2018, discontinued in the U.S. market 2023	HCL
Polatuzumab vedotin	Polivy	Genentech/Roche	CD79b	2019	DLBCL
Enfortumab vedotin	Padcev	Astellas/Seagen (now Pfizer)	Nectin-4	2019	Urothelial carcinoma
Trastuzumab deruxtecan	Enhertu	AstraZeneca/Daiichi Sankyo	HER2	2019	HER2+/HER2-low breast cancer, gastric cancer, NSCLC
Sacituzumab govitecan-hziy	Trodelvy	Gilead	Trop-2	2020	TNBC, urothelial carcinoma
Belantamab mafodotin-blmf	Blenrep	Glaxosmithkline	BCMA	2020; withdrawal in 2023; Reapproved in 2025	Multiple myeloma
Loncastuximab tesirine-lpyl	Zynlonta	ADC Therapeutics	CD19	2021	DLBCL
Tisotumab vedotin-tftv	Tivdak	Genmab/Seagen (now Pfizer)	Tissue factor	2021	Cervical Cancer
Mirvetuximab soravtansine-gynx	ELAHERE	ImmunoGen (now Glaxosmithkline)	FRα	2022	Ovarian Cancer
Datopotamab Deruxtecan	Datroway	AstraZeneca/Daiichi Sankyo	Trop-2	2025	HR+/HER2– Breast Cancer
Telisotuzumab vedotin	EMRELIS	AbbVie	c-Met	2025	NSCLC

FRα, foliate receptor alpha; AML, Acute Myeloid Leukemia; HL, Hogkin’s lymphoma, ALCL, Anaplastic large cell lymphoma; DLBCL, Diffuse large B-cell lymphoma; NHL, Non-Hodgkin’s lymphoma, ALL, Acute lymphoid leukaemia; HCL, Hairy cell leukemia; NSCLC, Non-small cell lung cancer; TNBC, Triple-negative breast cancer;.

Source: Tang et al.; Maiti et al.; https://www.fda.gov.

## 4 Immunotherapy and trials in Asia: Southeast Asia and Malaysia

Cancer immunotherapy has evolved from early approaches such as Bacillus Calmette-Guérin (BCG) in the 1990s to transformative modalities, including ICIs - notably anti-CTLA-4 and PD-1/PD-L1 therapies - as well as CAR-T and tumor-infiltrating lymphocyte (TIL) therapies. Together, these advances have redefined treatment paradigms and enabled durable responses across a broad spectrum of malignancies. In Asia and Southeast Asia, this progression is marked by a rapidly advancing innovation ecosystem and an increasingly dynamic landscape for clinical translation.

Asia has emerged as a major driver of immunotherapy innovation, propelled by strong government investment, expanding biotechnology sectors, and regulatory reforms. Japan marked a pivotal milestone with the 2014 approval of nivolumab (Opdivo) (142), positioning itself as an early regional leader in ICI adoption. China has since accelerated the pace of innovation, with the approval of multiple domestically developed anti-PD-1 agents, such as camrelizumab, tislelizumab, and toripalimab, beginning in 2018 (143–145). This growth reflects not only scientific advancement but also strategic national policies supporting biopharmaceutical development, streamlined clinical trial processes, and expedited regulatory pathways (143–145).

In Southeast Asia, the clinical uptake of immunotherapy has followed closely, with approvals of nivolumab and pembrolizumab between 2015 and 2018 in countries such as Singapore, Malaysia, and Thailand. While these milestones demonstrate increasing regional integration into global oncology practice, adoption remains heterogeneous. Key barriers include limited specialized infrastructure, high treatment costs, and disparities in access to biomarker testing and advanced therapeutics (59). These challenges are particularly significant given the region’s high prevalence of infection-associated cancers, including HCC (linked to HBV and HCV), cervical cancer (HPV), and NPC (EBV), which necessitate context-specific and biologically tailored immunotherapy strategies (1, 28).

**Figure 4:**
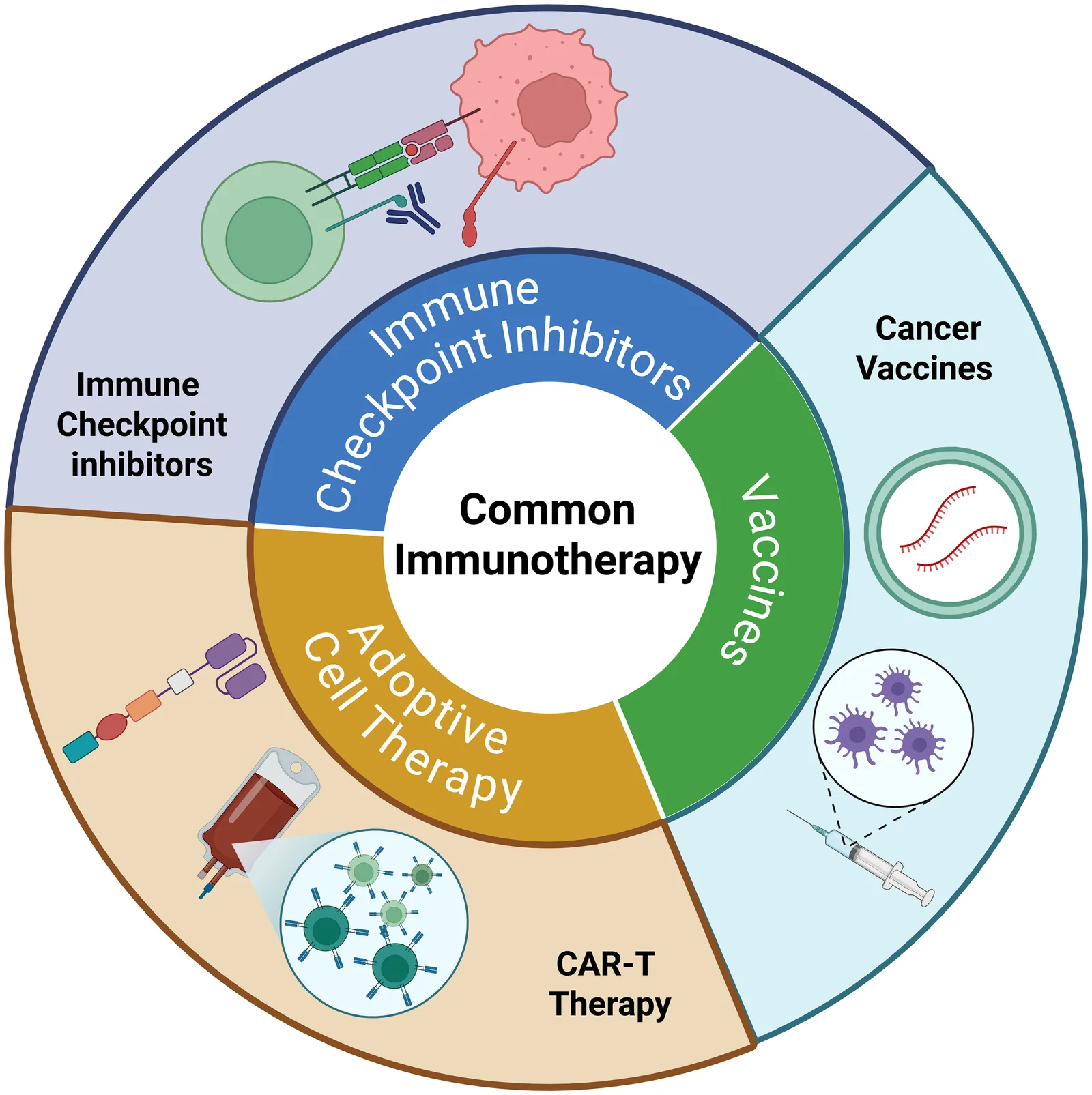
Immunotherapy modalities in Asia and Southeast Asia. Three main immunotherapy modalities in Asia and Southeast Asia. Created in BioRender. Phoon, Y. (2026) https://BioRender.com/a4gohib.

The regional therapeutic landscape is expanding beyond ICIs to encompass a broad spectrum of modalities, including CAR-T therapies and cancer vaccines (**Figure 4**). Additional emerging approaches, such as bispecific T-cell engagers (BiTEs), cytokine-based therapies and oncolytic viruses, are being actively investigated to address region-specific cancer burdens (146–149).

Despite this diversification, ICIs targeting PD-1/PD-L1 and CTLA-4 (e.g., nivolumab, pembrolizumab, camrelizumab, and toripalimab) remain the most clinically established, with demonstrated efficacy in malignancies such as melanoma, lung, and gastric cancers (39, 58, 122, 142–144). Their growing integration into national treatment guidelines, alongside regulatory approvals in countries including China, Japan, Singapore, and Malaysia, underscores a strong regional commitment to incorporating immunotherapy into standard oncology practice (122, 142, 143, 145, 150, 151).

Concurrently, CAR-T therapy development is advancing rapidly, with leading the region in the approval of locally developed products since 2021 (152). This progress is complemented by adoption in Japan and Singapore, where expedited regulatory pathways have facilitated timely clinical implementation (153, 154).

Crucially, the pace of innovation has been supported by evolving regulatory environments across the region. Agencies such as China’s NMPA, Japan’s PMDA, Singapore’s HSA, and Malaysia’s NPRA have introduced priority review and accelerated approval mechanisms to facilitate faster clinical translation of novel therapies. Despite this progress, disparities in regulatory capacity, healthcare infrastructure, and reimbursement systems continue to shape uneven access across countries (3, 59).

Overall, Asia and Southeast Asia are transitioning from adopters to active innovators in cancer immunotherapy, with clinical trials playing a central role in this shift. Sustained progress will depend on expanding regionally coordinated trial networks, increasing participation in early-phase and investigator-initiated studies, and enhancing cross-border collaboration and regulatory alignment to ensure more inclusive, efficient, and representative clinical research across diverse populations.

### 4.1 Immunotherapy clinical trial landscape

The immunotherapy clinical trial landscape in Asia and Southeast Asia has expanded rapidly, underscoring the region’s growing role in global oncology research. This growth is driven by an increasing number of early- and late-phase studies, greater participation in global trials, and strengthened regional research capacity and cross-border collaboration. Habr et al. further reported that approximately 46% of global clinical trials include sites in Asia, with China contributing substantially to this expansion (155), reflecting a robust and increasingly diverse pipeline of investigational therapies.

Over the past decade, Asia has experienced a substantial increase in clinical research activity, with approximately 126, 000 registered trials, representing a sevenfold rise in trial registrations. Japan accounts for the largest share (30.8%), followed by China, South Korea, and India, highlighting Asia’s emergence as a major global clinical development hub. Notably, the region’s annual growth rate in trial registrations has outpaced that of established regions such as the United States, Canada, the European Union, and Australia, indicating a clear global shift toward Asia in clinical research activity (156).

Within Southeast Asia, growth has been steady but more modest. Malaysia recorded an average annual growth rate of 8.4% in clinical trial activity between 2008 and 2017, reflecting gradual strengthening of its research capacity (156). **Supplementary Table 2** summarizes cancer immunotherapy clinical trials registered on ClinicalTrials.gov that included at least one study site in Malaysia. As of 2024, the World Health Organization (WHO) reports that the United States leads globally with nearly 190, 000 registered trials, followed by China (136, 000), India (74, 031), and Japan (65, 167). In Southeast Asia, trial numbers are significantly lower, including Thailand (12, 781), Singapore (4, 825), Malaysia (3, 664), the Philippines (2, 200), Indonesia (1, 533), Myanmar (127), Brunei (38), and Timor-Leste (29) (157). Although registry data provide an overview of research activity, they may not fully reflect the maturity or impact of cancer immunotherapy research across the region. Trial outcomes and publication data would complement this view in assessing research impact in Malaysia and the broader Southeast Asian region.

A survey of Asia-Pacific oncology institutions highlights strong engagement in immuno-oncology (IO) clinical research, with approximately 58% reporting active participation in immunotherapy trials and 25% conducting more than ten phase III studies over the past three years, reflecting increasing maturity in late-phase development (158). ICIs dominate both trial activity (81.8%) and clinical practice (88.6%), while CAR-T and TCR-T therapies remain less established, with participation rates of 22.7% and 2.3%, respectively (158). However, these findings are derived from an ASCO 2022 abstract and have not undergone peer review and remain as preliminary reported data.

CAR-T cell therapy development in Asia shows substantial heterogeneity, with China leading regional research (>342 trials by 2021), followed by Japan, while Southeast Asian participation remains constrained by infrastructure, regulatory, and manufacturing limitations (159). Likewise, although 124 CAR-NK trials had been registered globally by 2024, most remain early-phase and concentrated in China, with promising activity in hematologic malignancies but limited efficacy in solid tumors (160–162). Further discussion of CAR-T and CAR-NK therapeutic advances is provided in Section 5.

Collectively, these trends underscore Asia’s growing influence in advanced cellular immunotherapy, with China and the United States remaining the primary global hubs, while Asia more broadly emerges as a key driver of clinical innovation. However, ICIs remain the dominant and most widely established immunotherapy modality across the region. Sustained progress will depend on continued investment, strengthened clinical trial infrastructure, and progressive regulatory convergence to support broader access and translation of CAR-based therapies.

## 5 Innovation and clinical translation in Asia and Southeast Asia

Asia and Southeast Asia are emerging as key global hubs for clinical innovation and translation, driven by expanding research infrastructure, rising clinical trial activity, and a rapidly growing biotechnology sector. Countries including China, Japan, Singapore, South Korea, and Malaysia are making substantial investments in innovation, investigator-initiated research, and novel therapeutic platforms (159). This section examines the current innovation environment and regional strategies shaping biomedical advancement across Asia and Southeast Asia.

### 5.1 Artificial intelligence-driven digital health and innovation

The integration of artificial intelligence (AI) and big data analytics is accelerating immunotherapy development across Asia and Southeast Asia. Machine learning-based platforms facilitate early drug discovery by analyzing complex multi-omics datasets to identify novel immunotherapy targets and pathways, while machine learning approaches improve patient stratification using transcriptomic, proteomic, and immunologic profiles, enabling more precise and personalized treatment strategies. In parallel, AI applications in cancer screening, pathology, and radiology are enhancing diagnostic accuracy and supporting more informed immunotherapy decision-making (163, 164). For example, an AI tool developed through collaboration between institutions in Hong Kong and South Korea analyzes TILs in NSCLC to predict response to ICIs and optimize treatment selection (165). The increasing adoption of AI tools by academic centers and emerging biotech startups in the region reflects a broader shift toward data-driven immuno-oncology innovation (166).

In Malaysia, AI-enabled healthcare applications are being implemented across both public and rural settings, including telemedicine platforms, mobile screening tools such as MeMoSA^®^ for oral lesion triage with clinical-level performance (167, 168), and AI-assisted lung cancer screening initiatives in public hospitals. Notably, AstraZeneca Malaysia and the National Cancer Institute (Institut Kanser Negara, IKN) are collaborating under *Projek Saringan Awal Paru-Paru (SAPU)* as part of the Lung Ambition Alliance programme initated in 2021 to expand AI-assisted imaging in government healthcare facilities for early detection (169). Complementing this, academic-industry partnerships established in 2026 are advancing AI-based telemedicine solutions to improve access for rural and underserved populations, for example strategic collaboration between Asia Pacific University of Technology & Innovation (APU) and a Malaysian-owned private healthtech company (170). The successful implementation of digital health innovations requires supportive policy and regulatory frameworks. In Malaysia, national initiatives such as the Malaysia Digital Economy Blueprint (2021–2025) (171, 172), the National AI Roadmap (2021–2025) (172, 173), and regulatory sandbox programs introduced since 2018 (172, 174–176) have fostered a rapidly evolving digital health ecosystem (172). To facilitate the translation of emerging AI and digital health technologies, Malaysia has established regulatory sandboxes, including the National Regulatory Sandbox (2018) and the National Technology and Innovation Sandbox (NTIS, 2020), which provide structured environments for testing and validating innovative solutions (172, 174–176). These frameworks promote collaboration among academia, healthcare institutions, industry, and regulatory stakeholders to address technical, regulatory, and financial barriers. Their scope has been further expanded through the 2024 NTIS AI Sandbox Pilot Programme, involving collaborations between the Malaysian Research Accelerator for Technology and Innovation and NVIDIA (172, 177), a global leader in artificial intelligence and accelerated computing. Collectively, these initiatives provide a supportive ecosystem for the development and implementation of AI applications in immuno-oncology, including biomarker discovery, patient stratification, screening and clinical decision support.

Key challenges remain, including the need for standardized and interoperable clinical and multi-omics datasets to ensure reproducibility across institutions. Algorithmic bias is another concern, as models trained on non-representative datasets may perform suboptimally in the genetically diverse populations of Asia and Southeast Asia, potentially exacerbating health disparities. In addition, the digital divide, particularly unequal access to computational infrastructure and high-speed connectivity, continues to limit equitable implementation of AI-integrated immunotherapy in resource-limited and rural settings.

### 5.2 Biomarker for precision immunotherapy

The identification of robust, population-relevant biomarkers is essential for effective immunotherapy implementation in Asia and Southeast Asia, where biomarker-guided patient selection, response monitoring, and combination strategy design are central to overcoming therapeutic resistance (147, 178–181). However, many clinically validated biomarkers have been derived predominantly from Western populations and may not fully capture the genetic and environmental diversity of Asian cohorts.

To address this gap, countries such as Malaysia, Singapore, China, and South Korea are developing biobank networks and embedding biomarker research within clinical trial frameworks to generate region-specific datasets that support precision immunotherapy tailored to Asian populations (182–189). Standardization of biomarker use in clinical trials is also critical to improve data consistency and translational value. In this context, the Society for Immunotherapy of Cancer (SITC) Clinical Immuno-Oncology Network (SCION) has proposed a consensus panel of essential biomarkers for early-phase trials, including complete blood count with differential, lactate dehydrogenase (LDH), albumin, PD-L1 immunohistochemistry, microsatellite instability, and tumor mutational burden (TMB), to harmonize study design and enhance clinical comparability (190, 191).

Across Asia and Southeast Asia, evidence increasingly demonstrates substantial variability in immunotherapy-relevant biomarkers across populations, ethnic groups and tumor types, with important implications for precision oncology.

In NSCLC, large real-world Chinese cohorts report PD-L1 expression in approximately 49.5% of tumors with tumor proportion score (TPS) ≥1%, including 17.5% with high expression (≥50%), with enrichment in squamous histology and KRAS/MET/ROS1-altered tumors, and reduced expression in EGFR-mutant cancers (192). In parallel, a Malaysian NSCLC cohort (predominantly Chinese, 78%) shows a high EGFR mutation rate (57% vs 10–20% in Western populations) with substantial PD-L1 expression (~64%), while a multiethnic Malaysian cohort demonstrates no significant ethnic differences in PD-L1 distribution, but identifies driver mutations as independently associated with absent PD-L1 expression (193), highlighting the dominant role of tumor genomics over ethnicity alone. In other malignancies, comparable heterogeneity is observed. In triple-negative breast cancer, PD-L1 positivity in Malaysia is approximately 37.7%, with no significant association with ethnicity despite a predominantly Chinese cohort (55.8%) (194).

Apart from PD-LI, other biomarkers such as deficient mismatch repair (dMMR), TMB and loss of heterozygosity (LOH) are also widely used. In a Malaysian colorectal cancer cohort, the prevalence of dMMR is 9.9%, with lower rates in Chinese patients (6.2%) compared with Malays and Indians combined (14.8%), suggesting intra-national ethnic variation in immunotherapy-related biomarkers within the country (195). In oral squamous cell carcinoma, LOH at 3p occurs in 76% of Malaysian cases, representing an intermediate frequency between Japanese (60%) and Indian (96.2%) cohorts, reflecting regional differences in genomic instability (196).

Additionally, pan-cancer evidence indicates that TMB is not uniformly predictive across ancestries: TMB-high status is associated with better immunotherapy outcomes mainly in European ancestry patients, while African and Asian patients often do not derive the same benefit, suggesting reduced transferability due to genomic reference bias and differences in clinical response (197).

Compared with Caucasian cohorts, Asian breast cancers demonstrate selective differences: higher TP53 mutation frequency (~42.9% vs 30–35%), higher HER2 subtype prevalence, and increased immune infiltration (CD8+ T cells and macrophages). Clinically, TP53 mutations associate with poorer ER+ survival, while higher immune activity correlates with better outcomes, suggesting prognosis is driven more by immune context and key driver mutations than by mutational processes (198). Consistently, HR+/HER2− breast cancer in Asian populations (including Malaysia) shows high immune scores, with enrichment of immune cell types linked to adaptive immunity and increased abundance of cells involved in antigen presentation and recognition, suggesting enhanced immune activation and reinforcing the central role of immune contexture in disease heterogeneity (199).

Recently, the microbiome has gained increasing attention, with host-related factors such as the gut microbiome contributing to immunological variation. Malaysian cancer patients exhibit a shift from healthy Firmicutes-dominant, short-chain fatty acid (SCFA)-producing microbiota (e.g., Faecalibacterium, Roseburia, Bifidobacterium) toward Proteobacteria enrichment (e.g., Escherichia/Shigella, Klebsiella, Pseudomonas), indicating a pro-inflammatory microbial state that may influence immune response and treatment outcomes (200).

Most studies are derived from relatively small, single- or multi-center cohorts, often with uneven ethnic representation and limited stratification within populations and are frequently restricted to specific cancer types. This limits generalizability across broader cancer spectra and across diverse Asian subpopulations. In addition, variability in assay platforms, retrospective study designs, and selective biomarker testing further constrain direct comparison across studies.

Collectively, these findings underscore that biomarker landscapes in Asia and Southeast Asia are shaped by interactions between ethnicity, tumor genomics, immune microenvironment, and environmental exposures, highlighting the need for large-scale, prospective, and multi-omic region-specific validation studies to optimize precision immunotherapy strategies.

### 5.3 Cell-based immunotherapy advances

Cell-based immunotherapy represents a rapidly evolving frontier in cancer treatment, with CAR-T, CAR-NK, and TIL therapies driving advances in adoptive cellular immunotherapy across Asia and Southeast Asia. While China and Japan lead in regulatory approvals and clinical development, Southeast Asia remains in an early but growing phase, with emerging participation from Singapore and Malaysia through expanding clinical trial infrastructure, academic-industry collaborations, and early case-based implementations. Collectively, these trends highlight both rapid innovation and persistent regional differences in development, manufacturing capacity, and patient access across Asia. These comparisons are summarized in **Table 4**, providing a simplified overview of the cell therapy landscape across key Asian regions, including China, Japan, South Korea, Singapore, and Malaysia, which collectively represent the Asian ecosystem across different stages of cell therapy development.

**Table 4:** Regional overview of the cell therapy landscape in Asia.

Country	Category	Key strength	Main limitation	Outlook
China	Advanced, large-scale system	Broad capability with large scale capacity	Regulatory variation	Rapid expansion
Japan	Advanced	High-quality regulatory systems	Slow adoption	Steady growth
South Korea	Transitional, innovation-focused	Strong NK focus	Limited scale-up	Niche growth
Singapore	Transitional	Clinical and regulatory coordination	Small scale	Regional access role
Malaysia	Emerging	Growing clinical activity	Early-stage infrastructure	Gradual development

Represent the Asian ecosystem across different stages of cell therapy development.

#### 5.3.1 Chimeric antigen receptor T-cell therapy

CAR-T therapy development is advancing rapidly across Asia, with China leading in both innovation and regulatory approvals, driven largely by investigator-initiated studies (159). Notable examples include Legend Biotech’s ciltacabtagene autoleucel (cilta-cel), a BCMA-targeted CAR-T product approved by China’s NMPA (201), and CARsgen Therapeutics’ satricabtagene autoleucel (satri-cel, CT041), a Claudin18.2-targeting CAR-T therapy showing promising phase II results in gastrointestinal cancers (202). In contrast, Japan advances CAR-T development through a strong public-private ecosystem supported by the Japan Agency for Medical Research and Development (AMED) and industry partnerships, enabling clinical adoption of approved products such as Kymriah, Yescarta, Breyanzi, Abecma, and Carvykti (159).

In Southeast Asia, CAR-T activity remains early but increasingly visible. Singapore is expanding access through a hub-and-spoke model that supports regional CAR-T delivery and collaboration (154), while Malaysia is building capacity through academic-industry partnerships, participation in Phase II–III studies, and limited case-by-case clinical access via regulatory approval pathways, including programs such as Auxi Therapeutics-supported treatment models. These efforts are complemented by university-led trials in hematologic malignancies, including diffuse large B-cell lymphoma (DLBCL) and acute lymphocytic leukemia (ALL) (159), reflecting Malaysia’s emerging national engagement in CAR-T therapy. In parallel, Malaysia is strengthening its clinical trial infrastructure and biobank networks to support future cell-based research (203).

Parallel to CAR-T, CAR-NK cell therapy is emerging as a next-generation cellular immunotherapy platform alongside CAR-T, with global development accelerating since the first clinical trial in 2009 (NCT00995137). By 2024, 124 clinical trials had been registered, the majority in early Phase I, with approximately two-thirds conducted in China and one-third in the United States, while Southeast Asia remains minimally represented (160, 161). These studies span both hematologic malignancies (54.2%) and solid tumors (34.2%). In hematologic cancers, key targets include CD19, CD20, CD22, CD33, CLL1, and BCMA, alongside bispecific constructs such as CD33/CLL1 and CD33/FLT3. In solid tumors, commonly investigated targets include HER2, NKG2D ligands, PSMA, TROP-2, MUC1, Claudin18.2, ROBO1, and PD-L1. Early clinical findings show encouraging safety and preliminary efficacy in hematologic malignancies, whereas responses in solid tumors are generally characterized by tolerability and disease stabilization (160–162).

Regionally, China and South Korea are driving CAR-NK innovation. China leads early clinical development through multiple investigational products, including MUC1-, CD7-, and CD33-targeted CAR-NK constructs from PersonGen BioTherapeutics, as well as Robo1- and BCMA-directed biCAR-NK therapies developed by Asclepius Technology, all demonstrating encouraging safety and low incidences of graft-versus-host disease and severe toxicity (161, 204–206). South Korea has further advanced allogeneic CAR-NK strategies, including CD5-directed approaches under development by GC Cell Corporation (207) and early-phase clinical expansion such as GCC2005 (NCT06699771, 2025). In contrast, Southeast Asia remains in the early stages, with limited active CAR-NK programs, highlighting a regional development gap despite broader Asian leadership in the field.

#### 5.3.2 Tumor infiltrating lymphocyte therapy

Since its development by Rosenberg and colleagues in 1986 (208), TIL therapy has advanced over nearly four decades, culminating in its first FDA approval in 2024 with lifileucel (Iovance) for advanced melanoma (209). In metastatic melanoma, the most extensively studied and immunogenic indication, TIL therapy has demonstrated its greatest clinical success with ORRs ranging from 36–67%, complete response rates of up to 19%, and median overall survival of approximately 16–22 months. Durable long-term remissions exceeding 30 months have been observed in a subset of patients, underscoring its potential for sustained disease control (210–217). However, therapeutic efficacy remains constrained by the immunosuppressive TME, particularly T-cell exhaustion, prompting investigation of combination strategies with ICIs, which have shown improved ORR of 38.5–67% in advanced melanoma and activity in other solid tumors including HNSCC and cervical cancer (210, 218–220).

In Asia, TIL therapy is gaining increasing clinical interest, with China emerging as the clear leader in TIL clinical translation, driving a broad portfolio of early- and mid-phase studies across multiple solid tumor types. These include HCC, lung, head and neck, breast, gastric, colorectal, pancreatic, and esophageal cancers, as well as NPC and GBM, with emerging signals of activity. Many of these trials are investigator-initiated and conducted at major academic cancer centers, reflecting strong integration of translational immunology, large surgical oncology volumes, and expanding cell therapy manufacturing capacity. Additional early clinical trial sites in Singapore and South Korea are participating in Iovance-sponsored TIL therapy studies, primarily in melanoma, non-small cell lung cancer, and ovarian cancer. In contrast, most Southeast Asian countries, including Malaysia, remain at an early translational stage with limited or no registered TIL trials, although isolated case-based TIL manufacturing in Malaysia presented at the 2024 ASTCT–CIBMTR Tandem Meetings described a case of TIL manufacturing performed in Malaysia, highlighting both the feasibility of regional cell therapy development and the logistical and toxicity-related challenges associated with complex adoptive cell therapies (221). A summary of ongoing and completed TIL-related clinical trials across Asia and Southeast Asia is provided in **Supplementary Table 3**.

A major translational research initiative in Singapore supported by a US$6 million award is advancing liver and head and neck cancer programs through microbiome- and bile acid–based risk stratification to improve early detection of liver cancer in patients with fatty liver disease, alongside development of a cost-efficient, scalable TIL manufacturing platform for head and neck cancers. Collectively, these efforts reflect broader regional priorities in Asia to strengthen precision cancer screening and expand access to next-generation cell therapies for high-burden malignancies (222).

Japan has emerged as an important contributor to the development of TIL therapy through translational research and early clinical implementation. Initial Japanese studies demonstrated successful ex vivo expansion of TILs from melanoma, including rare subtypes such as mucosal and acral disease, but with marked phenotypic heterogeneity and no consistent correlation between tumor reactivity, CD8^+^ T-cell frequency, clinical parameters or immune checkpoint marker expression, suggesting a distinct and complex tumor-immune architecture (223). Subsequent feasibility study in ICI-refractory melanoma (UMIN000011431) confirmed manageable toxicity and variable clinical outcomes driven by factors such as MHC class I expression, CD4-dominant TILs, neoantigen burden, and suppressive MAPK, TGF-β, and Wnt/β-catenin pathways (224). Beyond melanoma, Japan has advanced structured clinical translation under PMDA oversight, including a Phase II trial of TIL therapy in recurrent cervical cancer (jRCTc031200283, TILCC trial) conducted with Keio University and REPROCELL (225, 226).

Collectively, these findings highlight that TIL therapy in Asia and Southeast Asia remains in an early translational phase, with growing but uneven clinical development across the region.

### 5.4 Translational research infrastructure

Asia’s rise in immunotherapy innovation is underpinned by strong research infrastructure, with China, Japan, and Singapore leading translational and early-phase clinical studies. These countries also play a central role in multinational collaborations, while navigating challenges related to cross-regulatory consistency, intellectual property protection, and ethical governance (163).

Within the region, Singapore and South Korea function as key early-phase clinical trial hubs, whereas countries such as Malaysia and Thailand are strengthening their capacity through investigator-initiated studies and biotechnology incubation programs. This diversification is broadening the regional research base and supporting more inclusive and representative clinical development (155, 157, 159).

Overall, Asia and Southeast Asia are positioned to become major contributors to global immunotherapy innovation. This progress is driven by the convergence of advanced research infrastructure, precision medicine approaches, and next-generation cellular therapies. However, continued investment in infrastructure, regulatory alignment, and cross-border collaboration will be essential to fully realize the region’s translational potential.

## 6 Challenges and gaps in immunotherapy implementation

The rising cancer burden in Asia and Southeast Asia presents significant challenges to the equitable delivery of modern oncology care, particularly for emerging therapies such as immunotherapy. Key barriers include limited healthcare resources, restricted access to screening and treatment, and the high cost and limited reimbursement of novel therapies. These challenges are compounded by geographic and socioeconomic disparities, diverse epidemiological and genetic profiles, underdeveloped research infrastructure and data systems, low public awareness, and complex regulatory environments. Collectively, these factors hinder the timely adoption and integration of immunotherapy, underscoring the need for targeted, region-specific strategies to improve access and implementation across diverse healthcare settings.

### 6.1 Infrastructure, cost, and access barriers

Access to immunotherapy in Asia and Southeast Asia is constrained by uneven healthcare infrastructure, with specialized oncology services largely concentrated in urban centers. High treatment costs, particularly for ICIs and CAR-T therapies, pose a major barrier, often exceeding the financial capacity of patients and public health systems in low- and middle-income settings. As a result, many patients face substantial out-of-pocket expenses, leading to treatment delays, interruptions, or abandonment. Limited reimbursement coverage, alongside prolonged health technology assessments and regulatory delays compared to Western markets, further restrict timely access (227, 228). In Southeast Asia, fragmented reimbursement frameworks underscore the need for more coordinated policies to support equitable and timely integration of immunotherapy.

In Malaysia, rising cancer care costs continue to strain patients and the healthcare system, particularly affecting low-income and rural populations. Geographic disparities, limited digital infrastructure, and shortages of specialized services hinder timely diagnosis and treatment in remote areas. Despite healthcare expenditure rising from MYR 23 billion in 2015 to MYR 29 billion in 2020, and sharply increasing to MYR 78 billion in 2021, public facilities still fall short of meeting the population’s growing health needs (163, 229). The high cost of advanced therapies, such as CAR-T therapy in Malaysia costs approximately USD 40, 000–50, 000 (MYR 170, 000–215, 000), further limits accessibility (159). Addressing these challenges will require increased public investment, expanded insurance coverage, improved distribution of specialized care, and stronger emphasis on prevention and early detection to ensure more equitable cancer care nationwide (34).

### 6.2 Health system disparities and immunogenomic heterogeneity

Southeast Asia faces pronounced geographic and socioeconomic disparities, resulting in unequal access to cancer care. Oncology services are largely concentrated in urban centers, leaving rural and remote populations underserved. Geographic barriers in archipelagic countries such as Indonesia and the Philippines, and mountainous regions such as Laos and Vietnam, further delay diagnosis and treatment, contributing to poorer outcomes (3).

Malaysia reflects similar inequities, with uneven healthcare distribution between Peninsular Malaysia and East Malaysia (Sabah and Sarawak). Cancer care is highly centralized, and workforce shortages persist: as of 2025, Malaysia has 184 oncologists for over 32 million people, below the WHO benchmark of 1 per 100, 000 population (34). Only 36.9% (68 out of 184) serve in Ministry of Health (MOH) facilities, despite public hospitals managing roughly 70% of national healthcare demand. In Sarawak, the largest state in Malaysia, just 12 oncologists serve approximately 2.5 million people (230, 231). Consequently, these workforce limitations contribute to delayed diagnosis and treatment, with nearly 60% of patients present at advanced stages (34). Based on WHO recommendations, Malaysia would require approximately 320 oncologists, indicating a current shortfall of 136 specialists (42.5%). Addressing this gap will require not only expanding specialist training pathways and oncology fellowship positions, but also implementing sustainable workforce retention strategies, including competitive remuneration, protected career development opportunities, research support, improved work-life balance, and incentives for service in underserved regions. Strengthening multidisciplinary oncology teams, leveraging tele-oncology networks, and creating clear professional advancement pathways may further enhance recruitment and long-term retention, particularly in geographically underserved areas.

These structural gaps are compounded by socioeconomic disparities, limiting equitable access to modern therapies, including immunotherapy.

Regionally, cancer patterns are shaped by infectious, genetic, environmental, and lifestyle factors. Infection-related cancers such as HCC, cervical cancer, and NPC remain highly prevalent. In Malaysia, Indigenous groups including the Orang Asli (aborigine) experience disproportionately higher cancer risks and reduced access to care (1, 3, 29, 163, 231). Genetic diversity further influences treatment response, particularly in immunotherapy. Compared with Western populations, Asian cohorts show greater immunogenomic variability. The Asian Immune Diversity Atlas (AIDA) has demonstrated significant differences in immune profiles across subgroups (232), yet most validated biomarkers remain Western-derived, limiting clinical applicability in Asian patients.

In Malaysia, rising cancer incidence is further driven by population aging, lifestyle factors (diet, inactivity, tobacco use), and environmental exposures. Collectively, these structural and biological disparities highlight the urgent need for equitable cancer prevention, early detection, and treatment across the region (3, 34).

### 6.3 Underrepresented cancers and populations

Several cancers with high incidence in Asia and Southeast Asia, such as biliary tract cancers (cholangiocarcinoma and gallbladder cancer) and oral cancers, remain underrepresented in immunotherapy clinical trials, limiting evidence for the development of effective, population-specific treatments (233). Although immunotherapy has expanded treatment options for these cancers - with ICIs, such as durvalumab and pembrolizumab, approved in combination with chemotherapy for biliary tract cancers, targeted therapies against FGFR2 and IDH1 alterations, and ICIs including pembrolizumab and nivolumab approved for recurrent or metastatic oral cancers - the clinical benefits have not been equitably translated into routine practice across the region (234).

Limited access to biomarker testing remains a major barrier to precision immunotherapy. Comprehensive molecular profiling, including next-generation sequencing (NGS), is unavailable or not routinely reimbursed in many Asian countries, including China, India, Indonesia, Malaysia, the Philippines, and Thailand, restricting the identification of actionable genomic alterations and patient selection for biomarker-directed therapies. Although Japan and Korea provide reimbursement under selected clinical circumstances, and Taiwan has recently introduced NGS reimbursement, access remains inconsistent across healthcare systems (234).

Even when actionable biomarkers are identified, reimbursement for immunotherapy and targeted therapies remains highly variable. While several Asian countries have approved ICIs and targeted therapies for biliary tract and oral cancers, public reimbursement is often limited or absent. In China, only selected immunotherapies and targeted agents are reimbursed, whereas most targeted therapies remain self-funded. In India, Indonesia, Malaysia, the Philippines, Thailand, and Taiwan, immunotherapies and targeted therapies are largely excluded from public insurance, resulting in substantial out-of-pocket costs. Similarly, reimbursement in Japan and Korea is restricted to selected therapies or indications, limiting equitable access despite comprehensive national health insurance systems (234).

In addition, rural populations and certain ethnic minorities are often underrepresented in clinical research, reducing the generalizability of findings and perpetuating healthcare disparities across the region (163). For example, in East Peninsular Malaysia, diverse indigenous groups, including Orang Asli subpopulations, experience distinct cancer patterns, with NPC being the most prevalent among the Iban population (231). Improving representation of these populations in clinical trials and expanding access to biomarker testing and reimbursed immunotherapies will be essential to achieve equitable implementation of precision oncology across Asia.

### 6.4 Cancer awareness and early detection gap

A major barrier to improving cancer outcomes in Southeast Asia is limited public awareness of cancer risks, symptoms, and the importance of early detection. Many individuals lack knowledge of warning signs and recommended screening practices, including mammography, Pap smears, and colonoscopy, contributing to late-stage diagnoses when treatment is less effective (235–237). Cultural stigma, fear, and misinformation further discourage timely medical consultation and delay diagnosis and care (237, 238). These challenges are compounded by low participation in cancer screening programs across the region, limiting early detection efforts (235, 239). Access to health education and screening services remains particularly constrained in rural and underserved communities (240). Strengthening public health education, integrating cancer awareness into primary care, and expanding access to screening services are essential to promote early diagnosis and reduce the cancer burden in Southeast Asia, including Malaysia.

### 6.5 Regulation and policy landscape

Regulatory frameworks for advanced therapies vary significantly across Asia and Southeast Asia, reflecting differing levels of maturity and national priorities. Japan’s PMDA has introduced the Sakigake designation to accelerate review of innovative therapies for serious conditions, while China’s NMPA has reformed its framework to incorporate real-world evidence and distinguish between levels of cell product manipulation. Singapore’s HSA applies a risk-based regulatory model under its Cell, Tissue and Gene Therapy Products (CTGTP) framework, and South Korea’s Ministry of Food and Drug Safety (MFDS) offers conditional approval pathways to enable early access based on Phase I/II data (163, 241).

In contrast, Malaysia remains in the early stages of developing a dedicated regulatory framework for advanced therapies. The MOH, through the NPRA and the National Committee for Clinical Research (NCCR), currently oversees these products primarily through case-by-case evaluations, with limited specific guidance on advanced therapies. For new biologics that address unmet clinical needs, the NPRA may grant conditionally approval based on Phase II clinical data, provided the product has already been registered with at least one Drug Control Authority reference agency, with the approval remaining valid for up to 2 years. Similarly, application for new indications of registered drugs may be considered case-by-case basis when comprehensive clinical data are lacking (242). Consequently, regulatory decisions frequently rely on assessments by established agencies such the FDA or EMA (243, 244).

In practice, regulatory approval in Malaysia typically occurs 1–2 years after FDA or EMA approval. Although newly approved therapies are often available promptly in private hospitals, access in the public healthcare system is frequently delayed, likely due to reimbursement and funding constraints (234). Strengthening regulatory capacity, defining clear approval pathways for advanced therapies, and aligning with regional harmonization efforts will be critical to supporting innovation and timely patient access.

One regional initiative to strengthen regulatory convergence across the 11 ASEAN member states (Brunei, Cambodia, Indonesia, Laos, Malaysia, Myanmar, the Philippines, Singapore, Thailand, Timor-Leste and Vietnam) is the ASEAN Pharmaceutical Product Working Group (PPWG). Through initiatives such as the ASEAN Common Technical Dossier (ACTD) and Joint Assessment Coordinating Group (JACG), the PPWG promotes regulatory reliance, reduces duplication of assessments, and supports more efficient access to innovative medicines, including advanced immunotherapies, while respecting national regulatory decisions (245).

Overall, regulatory heterogeneity across the region complicates cross-border collaboration, delays access to advanced therapies, and limits clinical integration. Harmonized clinical trial guidelines, regional regulatory working groups, and shared evaluation frameworks could streamline approvals and improve consistency in data submission. In addition, centralized regulatory databases and capacity-building initiatives would further support alignment, enhance efficiency, and promote regional cooperation in immuno-oncology development.

### 6.6 Post-marketing surveillance

Post-marketing surveillance (PMS) is essential for immunotherapy because pre-approval clinical trials are typically limited by small sample sizes, selected patient populations, and short follow-up durations, leading to under-detection of rare, delayed, and immune-related AEs (246, 247). Real-world pharmacovigilance is therefore critical for continuous benefit–risk evaluation after approval.

Marked differences exist in pharmacovigilance maturity across regions. Many Southeast Asian countries rely mainly on spontaneous adverse drug reaction (ADR) reporting systems with low reporting rates, manual case review, and limited use of quantitative signal detection methods, often constrained by variable infrastructure and regulatory capacity. In contrast, more established systems in countries such as the United States, United Kingdom, Japan, and South Korea integrate higher reporting volumes with advanced statistical and automated signal detection, enabling earlier and more robust safety signal identification. China has rapidly strengthened its pharmacovigilance framework but continues to face challenges in standardizing practices and generating high-quality real-world evidence (246).

Across Asia, pharmacovigilance is coordinated through national regulatory agencies and participation in the World Health Organization Programme for International Drug Monitoring, with increasing adoption of electronic reporting systems and patient registries. Japan uses a conditional approval system requiring intensive post-marketing data collection, while South Korea applies structured re-examination and post-marketing study requirements. Malaysia’s National Pharmaceutical Regulatory Agency oversees national pharmacovigilance through spontaneous ADR reporting from healthcare professionals, industry, and patients (248).

Common challenges across regions include underreporting of AEs, heterogeneous and incomplete data, limited integration of real-world evidence, and difficulty in establishing causality for complex immunotherapy toxicities. Fragmented databases and inconsistent reporting standards further hinder signal detection and data harmonization (246, 248).

Strengthening PMS for immunotherapy requires improved pharmacovigilance infrastructure, greater healthcare professional training and awareness, wider adoption of quantitative signal detection tools, and development of integrated national and international safety databases. Enhanced regulatory collaboration and harmonization are essential to improve early detection of immune-related AEs and support safer long-term use of immunotherapeutic agents.

## 7 Discussion

The cancer immunotherapy landscape is undergoing rapid transformation, with innovative treatments redefining cancer care both globally and across Asia and Southeast Asia. The region, including Malaysia, has made significant progress in clinical trial participation, cell and gene therapy development, and biomarker-driven research. However, several challenges persist, including managing genetic heterogeneity, improving equitable access to advanced therapies, navigating fragmented regulatory environments, and strengthening local data infrastructure and biobanking capacity. Advancing immunotherapy in this context requires a coordinated approach that integrates scientific innovation with practical strategies to address systemic gaps.

Regional coordination and standardization of clinical trial processes are critical to this effort. Initiatives such as World Immunotherapy Council (WIC)-Asia (163) and multicountry studies, including the zuberitamab study and the ASCOLT (Aspirin after completion of standard adjuvant therapy for colorectal cancer) trial spanning 74 sites across 12 Asia-Pacific countries, illustrate the feasibility of coordinated research across multiple sites. Additional momentum is provided by global initiatives such as Cure4Cancer and Project Orbis, while expansion of networks (e.g., inclusion of Australia in the WIC Australasia group) could further strengthen regulatory alignment and accelerate approvals (163, 249–251). These initiatives promote standardized trial management, improved data sharing, and consistent oversight, enhancing the quality and translational impact of immuno-oncology research. Despite progress in countries like Japan and China, disparities in regulatory maturity and infrastructure persist, limiting equitable trial participation and access to advanced therapies in lower-resource settings. Harmonizing regulatory frameworks across the region could accelerate approvals, streamline clinical development, and position Asia and Southeast Asia as leaders in global immunotherapy innovation.

Integration of AI, biomarker-guided personalized treatment strategies, and telemedicine offers practical solutions to optimize patient care and trial efficiency, particularly in geographically diverse settings such as Malaysia. Collectively, recent developments indicate that digital health innovations are transitioning from isolated pilot studies toward a more coordinated translational ecosystem supported by national policy frameworks and cross-sector collaboration. For example, the deployment of mobile-based screening tools such as MeMoSA^®^ demonstrates the feasibility of AI-assisted clinical triage in real-world settings in rural areas, supporting earlier specialist referral and decentralised care delivery. At the policy level, regulatory sandboxes such as the NTIS provide structured environments that enable testing and refinement of AI-driven healthcare solutions while facilitating regulatory adaptation. The growing collaboration among public healthcare institutions, universities, pharmaceutical companies, private healthtech stakeholders, and global AI industry leaders-including partnerships involving the IKN Malaysia, AstraZeneca Malaysia, APU, and NVIDIA-further highlights the emergence of a multi-stakeholder ecosystem capable of accelerating the development, evaluation, and implementation of AI-enabled healthcare solutions. These developments collectively suggest increasing integration of digital tools into clinical workflows, with potential to enhance diagnostic precision, patient stratification, and access to care. However, sustained impact will depend on continued improvements in infrastructure, greater consistency in regulatory approaches, and digital readiness across healthcare systems. Over the next decade, these technologies are expected to progressively integrate into routine oncology practice, contributing to improved treatment precision and reduced healthcare disparities.

Equally important are strategies addressing policy, workforce, education, and public awareness. Policy reforms, sustainable funding models, and public-private partnerships are essential to enhance affordability and access to high-cost therapies such as ICIs and CAR-T therapies. Fragmented reimbursement and insurance systems continue to constrain equitable access, prompting exploration of value-based pricing and outcome-linked risk-sharing models to enhance affordability and access (252, 253). In Malaysia, ongoing efforts to strengthen health technology assessment (HTA) frameworks aim to support cost-effective integration of immunotherapy into national formularies (254, 255). Workforce development through specialized training, fellowships, and interdisciplinary programs is essential to ensure safe delivery of advanced therapies and build expertise in precision oncology. In parallel, public education and community engagement initiatives can improve cancer awareness, promote early detection, and strengthen health literacy, particularly in underserved settings.

This review has several limitations. A substantial proportion of published studies and clinical trial data originate from China, reflecting its leading role in regional immunotherapy research and clinical development. Consequently, the available literature may underrepresent experiences, challenges, and implementation realities in other Asian and Southeast Asian countries. Furthermore, considerable heterogeneity in healthcare systems, regulatory frameworks, reimbursement policies, and access to immunotherapies may limit the generalizability of regional observations. Finally, given the rapidly evolving nature of cancer immunotherapy, particularly in cell-based therapies, biomarker-guided approaches, and emerging technologies, some findings may be superseded by newer evidence and therapeutic advances as the field continues to evolve.

Despite these limitations, this review provides a contemporary synthesis of the cancer immunotherapy landscape across Asia and Southeast Asia, highlighting key advances, implementation challenges, and emerging opportunities. Looking ahead, continued progress will depend on expanding access to immunotherapy, broader adoption of biomarker-guided precision medicine, and the strategic integration of AI and telemedicine into routine clinical practice. Regional collaboration, regulatory harmonization, and targeted investments in workforce development and healthcare infrastructure will be critical to translating scientific advances into accessible and effective cancer care. With sustained commitment, countries such as Malaysia are well positioned to contribute meaningfully to the global advancement of precision immuno-oncology and to improve outcomes in increasingly diverse patient populations, supported by growing clinical trial activity, expanding participation in immunotherapy and bsAbs studies, biosimilar-driven improvements in access, and emerging regulatory facilitation mechanisms, although further strengthening of policy frameworks and translational infrastructure will be essential to fully realize this potential. By consolidating fragmented evidence from a region that remains underrepresented in the global literature, this review aims to inform future research priorities, support policy development, and guide the sustainable implementation of immunotherapy across Asia and Southeast Asia.

## Glossary

ACTD: ASEAN Common Technical Dossier
ADR: Adverse drug reaction
AEs: Adverse events
AIDA: Asian Immune Diversity Atlas
AI: Artificial intelligence
ALL: Acute lymphocytic leukemia
AML: Acute myeloid leukemia
AMED: Agency for Medical Research and Development
ASCOLT: Aspirin after completion of standard adjuvant therapy for colorectal cancer
ASIRs: Age-standardized incidence rates
ASMRs: Age-standardized mortality rates
BCG: Bacillus Calmette-Guérin
BiTEs: Bispecific T-cell engagers
bsAbs: Bispecific antibodies
BTC: Biliary tract cancer
Btmb: Blood-based tumor mutational burden
CAR-T: Chimeric antigen receptor T-cell
CAR-NK: Chimeric antigen receptor natural killer
ccRCC: Clear cell renal cell carcinoma
CI: Confidence interval
CRC: Colorectal cancer
CRS: Cytokine release syndrome
CTGTP: Cell, Tissue and Gene Therapy Products
Dmmr: Deficient mismatch repair
DCR: Disease control rate
DLBCL: Diffuse large B-cell lymphoma
DOR: Duration of response
EBV: Epstein-Barr virus
EMA: European Medicines Agency
ESCC: Esophageal squamous cell carcinoma
EFS: Event-free survival
FDA: U.S. Food and Drug Administration
GEJ: Gastroesophageal junction
GBM: Glioblastoma
HBV: Hepatitis B virus
HCC: Hepatocellular carcinoma
HCV: Hepatitis C virus
HNSCC: Head and neck squamous cell carcinoma
HPV: Human papillomavirus
HR: Hazard ratio
HSA: Health Sciences Authority
HTA: Health technology assessment
ICIs: Immune checkpoint inhibitors
IO: Immuno-oncology
ITT: Intention-to-treat population
JACG: Joint Assessment Coordinating Group
LDH: Lactate dehydrogenase
LOH: Loss of heterozygosity
mAbs: Monoclonal antibodies
MDS: Myelodysplastic syndrome
MDSCs: Myeloid-derived suppressor cells
MFDS: Ministry of Food and Drug Safety
MHC: Major histocompatibility complex
MNCR: Malaysia National Cancer Registry Report
MOH: Ministry of Health
NCCR: National Committee for Clinical Research
NMPA: National Medical Products Administration
NPC: Nasopharyngeal carcinoma
NPRA: National Pharmaceutical Regulatory Agency
NSCLC: Non-small cell lung cancer
ORRs: Objective response rates
PFS: Progression-free survival
PMDA: Pharmaceuticals and Medical Devices Agency
PMS: Post-marketing surveillance
PPWG: Pharmaceutical Product Working Group
RCC: Renal cell carcinoma
SCFA: Short-chain fatty acid
SCION: SITC Clinical Immuno-Oncology Network
SCLC: Small cell lung cancer
SITC: Society for Immunotherapy of Cancer
TC: Tumor cell
TCEs: T-cell engagers
TCR: T-cell receptor
TIL: Tumor infiltrating lymphocyte
TME: Tumor microenvironment
TNBC: Triple-negative breast cancer
TPS: Tumor proportion score
Tregs: Regulatory T cells
WIC: World Immunotherapy Council
WHO: World Health Organization.
